# Knowledge and correlates of use of safer conception methods among HIV-infected women attending HIV care in Uganda

**DOI:** 10.1186/s12978-019-0717-6

**Published:** 2019-05-29

**Authors:** Violet Gwokyalya, Jolly Beyeza-Kashesya, John Baptist Bwanika, Joseph K. B. Matovu, Shaban Mugerwa, Jim Arinaitwe, Dickson Kasozi, Justine Bukenya, Rosemary Kindyomunda, Glenn J. Wagner, Fredrick E. Makumbi, Rhoda K. Wanyenze

**Affiliations:** 1 Makerere University School of Public Health, Kampala, Uganda; 2 Department of Obstetrics and Gynecology, Mulago National Referral Hospital, Kampala, Uganda; 3 Ministry of Health, Kampala, Uganda; 4 UNFPA, Kampala, Uganda; 5 RAND Corporation, Santa Monica, CA USA

**Keywords:** Safer conception methods, Knowledge, HIV, Uganda, Reproductive health

## Abstract

**Background:**

Many people living with HIV would like to have children but family planning (FP) services often focus on only contraception. Availability of safer conception services is still very low in most low income countries. In this study we assessed the knowledge and use of safer conception methods (SCM) among HIV infected women in HIV care in Uganda to inform integration of safer conception in existing FP services.

**Methods:**

Data were accrued from a nationally representative cross-sectional survey of 5198 HIV+ women aged 15–49 years from 245 HIV clinics in Uganda. Knowledge and use of safer conception methods and associated factors were determined. The measure of association was prevalence ratio (PR) with corresponding 95% confidence intervals, obtained using a modified Poisson regression via generalized linear models. All the analyses were conducted using STATA version 12.0.

**Results:**

Overall knowledge of any safer conception method was 74.1% (3852/5198). However only 13.2% knew 3 to 4 methods, 18.9% knew only 2 methods and 42% knew only one method. Knowledge of specific SCM was highest for timed unprotected intercourse (TUI) at 39% (*n* = 2027) followed by manual self-insemination (MSI) at 34.8% (*n* = 1809), and pre-exposure prophylaxis (PrEP) at 24.8% (*n* = 1289). Knowledge of SCM was higher in the Eastern region (84.8%, *P* < 0.001), among women in HIV-discordant relationships (76.7%, *p* < 0.017), and those on ART (74.5%, *p* < 0.034). Overall, 1796 (34.6%) women were pregnant or reported a birth in the past 2 years—overall use of SCM in this group was 11.6% (209/1796). The odds of use of SCM were significantly lower in Kampala [adj. PR = 0.489(0.314, 0.764)] or Eastern region [adj.PR = 0.244; (0.147, 0.405)] compared to Northern region. Higher odds of SCM use were associated with HIV status disclosure to partner [adj.PR = 2.613(1.308, 5.221)] and sero-discordant compared to HIV+ concordant relationship [adj.PR = 1.637(1.236, 2.168)]. Pre-existing knowledge of any one SCM did not influence SCM use.

**Conclusions:**

Knowledge and use of SCM among HIV+ women in care is low. Efforts to improve HIV status disclosure, integration of safer conception into FP and HIV services and regional efforts to promote sensitization and access to safer conception can help to increase uptake of safer conception methods.

**Electronic supplementary material:**

The online version of this article (10.1186/s12978-019-0717-6) contains supplementary material, which is available to authorized users.

## Background

Uganda has one of the youngest and most rapidly growing populations in the world; its total fertility rate is among the world’s highest at 5.8 children per woman [1] yet with a high HIV prevalence of 6.2% [2]. Between 14 and 73% of people living with HIV (PLHIV) want to have children [3–5]. About 40% of HIV-infected women become pregnant post HIV diagnosis [6, 7] and over half (57%) of these pregnancies are planned [6]. In addition approximately half of HIV-affected couples in Uganda are in discordant relationships [8] and a significant proportion (60%) of new infections occurs in such relationships [9, 10]. HIV negative partners in serodiscordant relationships in which pregnancy occurs have nearly double the risk of HIV acquisition compared to their counterparts in the absence of pregnancy [11]. Antiretroviral therapy (ART) greatly reduces the transmission risk associated with childbearing, [12] especially when one achieves viral suppression [13] but many PLHIV are either not yet diagnosed or are diagnosed but not yet in care, while some of those on ART may not have achieved viral suppression for various reasons. According to the Global UNAIDS 2017 report, only 60% of all HIV positive people on treatment in Uganda have achieved viral suppression [13]. Thus, use of safer conception methods (SCM) such as manual self-insemination (MSI), timed unprotected intercourse (TUI) and sperm washing (for those who can afford) remains very relevant in this context to minimize the risk of sexual transmission during attempts to conceive [14].

International reproductive guidelines shifted a decade ago from recommending avoidance of pregnancy to recognizing conception and parenting as realistic options and a fundamental reproductive right for PLHIV and their partners [15]; encouraging detailed pre-conceptual counseling on all their conception options. Since 2001, the US Centers for Disease Control and Prevention (US CDC) has also encouraged information and support for HIV-affected couples who want to explore their reproductive options [16]. The Society of Obstetricians and Gynaecologists of Canada [17] and the South African HIV Clinicians Society [18] provided guidelines for safer conception with a range of strategies. These guidelines include: (i) fostering discussions between the clinicians and the clients on issues of childbearing; (ii) identifying the fertility desires of HIV-infected women and men, with discussions on contraceptive strategies for couples that do not desire a pregnancy; and (iii) management of HIV affected individuals and couples who desire a pregnancy, with emphasis on the management of HIV disease and co-morbidities before attempting conception, and offering specific conception strategies for HIV sero-concordant positive and sero-discordant couples. However, these guidelines have not yet been adopted by governments in resource-limited settings or incorporated as standard of care and as such the use of safer conception methods is still very low in most countries in sub-Saharan Africa and Uganda in particular [5, 19, 20]. Barriers to implementation of these guidelines include patient and provider stigma and lack of patient counseling regarding childbearing, lack of safer conception skills among health providers, and cultural barriers [5, 19].

PLHIV and their providers rarely discuss childbearing prior to pregnancy [21], resulting in a lost opportunity to promote safer conception, as well as contraception. In most sub-Saharan African (SSA) countries [20] including Uganda [5], most PLHIV (60–80%) do not discuss their fertility intentions with providers; providers have openly discouraged (and even scolded) clients from having children because of transmission and mortality risks [22–25] while clients avoid talking with providers about their pregnancy needs due to perceived provider stigma and internalized stigma. Comprehensive knowledge and counseling about safer conception has remained low, and thus affected the use of safer conception services among HIV-infected clients [20]. A study done among 48 clients attending a specialized HIV care centre in Uganda found that 61% had heard of one or more methods to reduce risk during conception with knowledge being highest with TUI (60%) and least with sperm washing (23%), but only 37% (*n* = 18) were able to describe any of the methods [19]. Majority of clients considered safer conception methods as abnormal and expressed concerns such as risk for HIV transmission, which presumably affects the uptake of these methods. Similarly, in a study of SCM in a sample of 400 Ugandan HIV clients in committed heterosexual relationships who have intentions to have a child, just over half knew that MSI (53%) and TUI (51%) reduced transmission risk during conception, and 15% knew of sperm washing and pre-exposure prophylaxis [26]. Only 12% had used timed unprotected intercourse while trying to conceive, but none had used manual self-insemination or sperm washing [27]. However, most prior studies have been small and the findings may not fully represent the large HIV population in care in Uganda. We therefore assessed knowledge, and correlates of use of SCM in a nationally representative sample of HIV infected women in the five geographical regions of Uganda to inform integration of safer conception into FP and sexual and reproductive health (SRH) services for HIV affected clients.

## Methods

### Study design, population and sites

Data are accrued from a study of 5198 HIV+ women from a nationally representative sample of 245 private and public HIV care facilities across five geographical regions in Uganda; Northern, Eastern, Western, Central and Kampala. The purpose of the study was to assess sexual and reproductive health (SRH) and family planning (FP) service integration, delivery models, and uptake of HIV care services among HIV-infected individuals in care. The health facilities were selected across various levels of health care delivery in Uganda including hospitals and health centers (HCIV, HCIII, and HCII) with chronic HIV care/treatment clinics. Health centres are lower level service delivery units categorized according to the size of the population served, staffing levels and type of services offered, the lowest being a health centre one (HC I) and the highest a health centre four (HC IV).

### Sampling

A two-stage sampling process was used. In the first stage, a sampling frame with a list of accredited HIV care facilities was used to randomly select an equal number of facilities in each region. The second stage of sampling was selection of study participants at the facility level. All HIV+ women aged 15–49 years who presented at the selected clinics for HIV care on the interview days were registered on their service sign-in daily attendance sheets. Systematic sampling was then conducted to randomly select the required number of eligible women from the daily attendance lists. After sampling, a brief screening tool was used to assess eligibility including age (only those aged 15–49 years were eligible for this study) and being sexually active (only those who had had sexual intercourse at least once within 12 months were eligible). Eligible participants provided written informed consent process before conducting the full interview.

### Sample size

The sample size calculation was based on unmet need for FP as the main outcome of the study assuming a 30% unmet need for FP among HIV+ women in care, 3.6% margin of error, 5% type-I error rate, a design effect of 1.5 and non-response of 10%. Based on these assumptions, we obtained a sample size of 5185 across the five geographical regions (i.e. 1037 respondents per region). Approximately 20 participants were selected from each facility with the exception of the highest volume facilities in Kampala with > 5000 clients in HIV care where 30–50 participants were randomly selected per site. Kampala has fewer but larger volume dedicated HIV facilities compared to the other regions. Overall, participants were selected from 52 facilities in each region except Kampala where participants were drawn from 37 ART accredited facilities.

### Data collection methods and procedures

Using an interviewer-administered structured questionnaire, data were collected on general knowledge about safer conception methods namely; TUI, MSI, PrEP for HIV-uninfected partners, and sperm washing. Women responded to a series of questions on whether or not they knew about SCM, and if they used any of the SCM singly or in combination for those pregnant or reporting childbearing in the past 2 years. Other safer conception questions included questions on strategies to reduce HIV transmission risk when planning for conception; for example, early initiation of ART for HIV-infected individuals, and having an undetectable viral load. Women also responded to questions on reproductive history; children ever been born alive, desire for a (more) child (ren) and timing of the next pregnancy. Data collection was conducted between September and November 2016 by a well-trained and experienced team of 30 interviewers. Pretesting of tools was conducted to check the suitability of various aspects of the questionnaires such as the translation, skip procedures and filtering questions, and modifications were done prior to actual data collection. All data collection tools were translated into the common languages of the selected regions.

### Measures

The primary outcome variable was reported knowledge of safer conception methods, or use of any SCM. Knowledge of safer conception methods was assessed using a series of general awareness and methods-specific statements that were read to the respondents with responses coded as “True”, “False” or “Don’t Know”. General awareness statements sought to explore respondents knowledge on the concept of ‘safer conception’ in general (e.g. “*there are ways that can make conception with an HIV negative partner safer”)* while methods-specific questions aimed to explore respondents’ knowledge of specific SCM (e.g. *TUI –* “*having unprotected sex during the few days each month when the woman is most fertile will help to limit the risk of HIV transmission to an uninfected partner”)*. Respondents who answered “true” to any of the general or methods-specific statements were presumed to have knowledge of safer conception in general and SCM in particular. Individuals who knew SCM were grouped into those who knew: a) 3–4 methods (designed as having a “high knowledge” of SCM); b) only two methods (designated as having “moderate knowledge”) or c) only one method (designated as having “poor knowledge”). Use of safer conception methods was determined among women who reported a birth within the last 2 years or were pregnant. A woman was classified as a user of SCM if she reported use of any of the SCM (TUI, MSI, PrEP for the HIV uninfected partner for those who are in sero-discordant relationships, and sperm washing) to reduce the risk of HIV transmission while trying to conceive a child with their partner.

### Data management and analysis

We conducted descriptive analyses to describe the socio-demographic characteristics of the women in the sample, the HIV diagnosis and treatment status, partner characteristics including their HIV status, knowledge and use of SCM. The associations between knowledge or use of SCM, and woman’s characteristics were assessed with prevalence ratio (PR) as the measure of association. PR was obtained using a “modified” Poisson regression model via a generalized linear model with family as Poisson and link as log and robust standard errors. All analysis used Stata version 12. In the bivariate to determine independent factors associated with the outcomes, the models included the following covariates; age, region, religion, health facility level and ownership, education level, wealth quintile, marital status, ART status of client and duration on ART, HIV disclosure status to partner, partner’s HIV status, partner’s ART status if also HIV positive, and number of biological children. Variables that were significant at a level of 5% significance or important from other previous studies were included in multivariable analysis.

## Results

### Participants’ characteristics

Table 1 shows the characteristics of 5198 women that were considered for this analysis. The mean (SD) age was 32.5 (7.2) years, and 85.6% were either married (59.2%, *n* = 3079) or in a relationship (26.4%, *n* = 1371). Enrolment on ART was nearly universal (96.6%, *n* = 5022) with a median (IQR) period of 3 (2, 6) years, 57.4% (*n* = 2985) were in sero-concordant relationships and 18.4% (*n* = 954) in discordant relationships, the rest did not know their partners’ HIV status. Close to a quarter (23%, *n* = 1168) of women in this study were either pregnant (5.6%) or wanted a child (17.5%) and another 28% (*n* = 1422) did not use a condom on their last sexual encounter because their partner wanted a child. Among those who wanted to conceive, 18% (*n* = 122) had HIV negative partners. HIV serostatus disclosure to a sexual partner was 84.5% (*n* = 4362); significantly higher (*p* < 0.001) in HIV sero-concordant (97.9%; *n* = 2922) than in HIV-discordant (89%; *n* = 849) relationships and those with partner of unknown HIV status (46.9%, *n* = 590).

**Table 1:** 15–49-year-old HIV positive women in care by selected characteristics

Characteristic	Total, *N* = 5198%	Region				
		Kampala, *N* = 1048 (%)	Central, *N* = 1032 (%)	Eastern, *N* = 1034 (%)	Western, *N* = 1039 (%)	Northern, *N* = 1045 (%)
Age						
15–19	103 (2.0)	14 (1.3)	22 (2.1)	15 (1.5)	25 (2.4)	27 (2.6)
200–24	657 (12.6)	150 (14.3)	132 (12.8)	115 (11.1)	147 (14.1)	113 (10.8)
25–29	1147 (22.1)	273 (26.0)	209 (20.3)	215 (20.8)	238 (22.9)	212 (20.3)
30–39	2254 (43.4)	452 (43.1)	449 (43.5)	470 (45.5)	437 (42.1)	446 (42.7)
40–49	1037 (20.0)	159 (15.1)	220 (21.3)	219 (21.2)	192 (18.5)	247 (23.6)
Health Facility Level^a^						
Hospital	1556 (29.9)	224 (21.4)	316 (30.6)	284 (27.5)	343 (33.0)	389 (37.2)
HC IV	1540 (29.6)	85 (8.1)	273 (26.5)	405 (39.2)	400 (38.5)	377 (36.1)
HC III	1542 (29.7)	417 (39.8)	366 (35.5)	244 (23.6)	256 (24.6)	259 (24.8)
HC II	416 (8.0)	288 (27.5)	1 (0.1)	81 (7.8)	38 (3.7)	8 (0.8)
Private health unit	112 (2.2)	34 (3.2)	58 (5.6)	20 (1.9)	0 (0.0)	0 (0.0)
Others	32 (0.6)	0 (0.0)	18 (1.7)	0 (0.0)	2 (0.2)	12 (1.1)
Religion						
Catholic	2136 (41.1)	354 (33.8)	441 (42.7)	315 (30.5)	415 (39.9)	611 (58.5)
Anglican / Protestant	1616 (31.1)	284 (27.1)	273 (26.5)	370 (35.8)	410 (39.5)	279 (26.7)
Moslem	663 (12.8)	199 (19.0)	150 (14.5)	183 (17.7)	53 (5.1)	78 (7.5)
Pentecostal / Born Again / Evangelical	662 (12.7)	188 (17.9)	137 (13.3)	155 (15)	115 (11.1)	67 (6.4)
Others	121 (2.3)	23 (2.2)	31 (3.0)	11 (1.1)	46 (4.5)	10 (1.0)
Marital status						
Never married	107 (2.1)	25 (2.4)	7 (0.7)	23 (2.2)	32 (3.1)	20 (1.9)
In relationship but not married	1371 (26.4)	349 (33.3)	362 (35.1)	172 (16.6)	163 (15.7)	325 (31.1)
Married	3079 (59.2)	596 (56.9)	555 (53.8)	690 (66.7)	672 (64.7)	566 (54.2)
Divorced/separated	412 (7.9)	60 (5.7)	77 (7.5)	85 (8.2)	105 (10.1)	85 (8.1)
Widowed	229 (4.4)	18 (1.7)	31 (3)	64 (6.2)	67 (6.4)	49 (4.7)
Education^b^						
No education	726 (14.0)	65 (6.2)	143 (13.9)	145 (14.0)	157 (15.1)	216 (20.7)
Primary	2924 (56.3)	449 (42.8)	606 (58.7)	565 (54.6)	653 (62.8)	651 (62.3)
Secondary	1381 (26.6)	445 (42.5)	260 (25.2)	303 (29.3)	221 (21.3)	152 (14.5)
More than secondary	156 (3.0)	89 (8.5)	21 (2)	21 (2.0)	7 (0.7)	18 (1.7)
Missing	11 (0.2)	0 (0.0)	2 (0.2)	0 (0.0)	1 (0.1)	8 (0.8)
Wealth quintile						
Lowest	1054 (20.3)	22 (2.1)	170 (16.5)	282 (27.3)	230 (22.1)	350 (33.5)
Second	1026 (19.7)	35 (3.3)	221 (21.4)	244 (23.6)	224 (21.6)	302 (28.9)
Middle	1041 (20.0)	64 (6.1)	231 (22.4)	253 (24.5)	249 (24.0)	244 (23.3)
Fourth	1039 (20.0)	328 (31.3)	228 (22.1)	146 (14.1)	239 (23.0)	98 (9.4)
Highest’	1038 (20.0)	599 (57.2)	182 (17.6)	109 (10.5)	97 (9.3)	51 (4.9)
Owns a radio						
No	1822 (35.1)	346 (33.0)	294 (28.5)	399 (38.6)	354 (34.1)	429 (41.1)
Yes	3376 (64.9)	702 (67.0)	738 (71.5)	635 (61.4)	685 (65.9)	616 (58.9)
Owns a Cell phone						
No	991 (19.1)	58 (5.5)	187 (18.1)	240 (23.2)	243 (23.4)	263 (25.2)
Yes	4207 (80.9)	990 (94.5)	845 (81.9)	794 (76.8)	796 (76.6)	782 (74.8)
On antiretroviral therapy						
No	176 (3.4)	51 (4.9)	6 (0.6)	26 (2.5)	52 (5.0)	41 (3.9)
Yes	5022 (96.6)	997 (95.1)	1026 (99.4)	1008 (97.5)	987 (95.0)	1004 (96.1)
Duration on ART (Years)						
< 1	562 (11.3)	166 (16.8)	128 (12.5)	87 (8.7)	100 (10.2)	81 (8.1)
1	570 (11.4)	127 (12.8)	129 (12.6)	105 (10.5)	110 (11.2)	99 (9.9)
2	739 (14.8)	140 (14.1)	157 (15.4)	138 (13.8)	154 (15.7)	150 (15.1)
3+	3118 (62.5)	558 (56.3)	607 (59.5)	670 (67)	617 (62.9)	666 (66.9)
Partner’s HIV Status						
Positive	2985 (57.4)	468 (44.7)	539 (52.2)	593 (57.4)	585 (56.3)	800 (76.6)
Negative	954 (18.4)	243 (23.2)	178 (17.3)	201 (19.4)	213 (20.5)	119 (11.4)
Don’t know	1259 (24.2)	337 (32.2)	315 (30.5)	240 (23.2)	241 (23.2)	126 (12.1)
Disclosed HIV status to partner						
No	800 (15.5)	301 (29.0)	214 (20.8)	114 (11.1)	133 (12.9)	38 (3.7)
Yes	4362 (84.5)	736 (71.0)	813 (79.2)	914 (88.9)	902 (87.1)	997 (96.3)
Number of biological children						
0	1342 (25.8)	267 (25.5)	318 (30.8)	207 (20.0)	330 (31.8)	220 (21.1)
1	588 (11.3)	193 (18.4)	104 (10.1)	93 (9.0)	104 (10.0)	94 (9.0)
2	852 (16.4)	220 (21.0)	164 (15.9)	133 (12.9)	176 (16.9)	159 (15.2)
3	764 (14.7)	169 (16.1)	132 (12.8)	157 (15.2)	147 (14.1)	159 (15.2)
4+	1652 (31.8)	199 (19.0)	314 (30.4)	444 (42.9)	282 (27.1)	413 (39.5)

^a^Health facility level refers to the categorization of levels of health care according to the size of the population served, the staffing and type of services offered, **HC II** serves up to 5000 people and only offering out-patient services to hospital that offers specialized care in addition to in and out-patient services and surgical operations.

^b^Education categories refer to the highest level of education attended, whether or not that level was completed.

### Knowledge of safer conception methods and ways to reduce HIV transmission risk

Overall 80.2% (*n* = 4262) had general knowledge of safer conception methods and 74.1% (*n* = 3852) knew at least one SCM. Of those that knew at least one SCM, 42% (*n* = 2183) knew only one method (had low knowledge), 18.9% (*n* = 986) knew only two methods (had moderate knowledge) and 13.2% (*n* = 685) knew three to four SCM (had high knowledge). Table 2 shows that knowledge of any one SCM was higher in the Eastern region (84.8%, *n* = 877, *P* < 0.001), among women in HIV-discordant relationships (76.7%, *n* = 731, *p* < 0.017), those in a higher wealth status (*p* < 0.003), and among those on ART (74.5%, *n* = 3741, *p* < 0.034). Knowledge of specific SCM was lower for MSI (35%, *n* = 1809) compared to TUI (39.0%, *n* = 2027), Table 3. TUI knowledge was higher in Central (47%) and Kampala (46%) regions while MSI was highest in Eastern region where 63% of women knew that a man could ejaculate in a condom or container and manually insert the semen into a woman’s vagina.

**Table 2:** Knowledge of at least one method of Safer Conception by selected characteristics

Among all women age 15–49, in HIV care, the percentage who answered ‘true’ to at least one knowledge statement on safer conception strategies, by background characteristics				
	% Not Knowledgeable	% Knowledgeable	Number	*P*-Value
Age				
15–19	39.8	60.2	103	0.014
20–24	27.4	72.6	657	
25–29	24.5	75.5	1147	
30–39	25.6	74.4	2254	
40–49	25.7	74.3	1037	
Region				
Kampala	23.0	77.0	1048	< 0.001
Central	22.7	77.3	1032	
Eastern	15.2	84.8	1034	
Western	34.9	65.1	1039	
Northern	33.4	66.6	1045	
Religion				
Catholic	27.4	72.6	2136	0.153
Anglican/Protestant	25.6	74.4	1616	
Moslem	22.0	78.0	663	
Pentecostal/Born again/Evangelic	25.4	74.6	662	
SDA^a^	26.0	74.0	104	
Other	23.5	76.5	17	
On ART				
No	32.9	67.1	161	0.034
Yes	25.5	74.5	5022	
Duration on ART (Years)				
< 1	27.7	72.3	559	0.401
1	26.3	73.7	570	
2	26.3	73.7	739	
3	24.7	75.3	3118	
Currently Married				
No	26.9	73.1	2119	0.136
Yes	25.1	74.9	3079	
Marital status				
Never married	28.0	72.0	107	0.572
In relationship but not married	26.5	73.5	1371	
Married	25.1	74.9	3079	
Divorced/Separated	28.4	71.6	412	
Widowed	26.2	73.8	229	
HIV Disclosure to Partner				
No	26.5	73.5	800	0.634
Yes	25.7	74.3	4362	
HIV Status of Partner				
Positive	25.6	74.4	2985	0.017
Negative	23.3	76.7	954	
DK/DR	28.5	71.5	1259	
Education				
No education	27.4	72.6	726	0.574
Primary	26.1	73.9	2924	
Secondary	25.0	75.0	1381	
More than secondary	21.8	78.2	156	
Missing	27.3	72.7	11	
Wealth Quintile				
Lowest	28.9	71.1	1054	0.003
Second	28.6	71.4	1026	
Middle	25.1	74.9	1041	
Fourth	23.3	76.7	1039	
Highest	23.4	76.6	1038	
Level Health Facility				
Hospital	26.5	73.5	1556	0.008
Health Center IV	26.5	73.5	1540	
Health Center III	26.2	73.8	1542	
Health Center II	23.6	76.4	416	
Private Health Unit	10.7	89.3	112	
Others	30.8	69.2	26	
Ownership				
Government/Public	26.1	73.9	3575	0.784
Mission/NGO/Private not for prof	25.3	74.7	1549	
Private for profit	23.9	76.1	71	
Total	25.9	74.1	5198	

^a^SDA refers to people of the Seventh Day Adventist Faith

**Table 3:** Knowledge of specific safer conception methods

Among all women age 15–49, in HIV care, the percentage who answered ‘true’ to knowledge statements on specific safer conception strategies, by background characteristics						
Background characteristic	Women knowledgeable that:					Number of women
	Having an “undetectable” amount of HIV Virus reduces risk of HIV transmission	Having the man ejaculate into a condom or container and then manually inject the semen into the woman’s vagina is a way to reduce risk of HIV transmission if the man is HIV negative (MSI)	Having unprotected sex during the few days each month when the woman is most fertile will help to limit the risk of HIV transmission to an uninfected partner (TUI)	There is technology available that can cleanse a man’s sperm or semen of the HIV virus (Sperm washing)	HIV medications can be taken by an HIV-negative (or unknown status) partner that will reduce their risk of getting infected by their HIV+ partner (PrEP)	
Age						
15–19	26.7	24.8	31	16.8	27.6	103
20–24	32.2	34.2	37.5	16.3	22.5	657
25–29	35.1	36.4	40.1	15.6	26.4	1147
30–39	34.3	35.7	39.7	19	24.4	2254
40–49	33.2	32.6	38.1	18.8	25.1	1037
Marital status						
Never married	34.9	37.7	32.1	18.9	29.5	107
In relationship but not married	36.7	32.9	38.1	17.3	23.2	1371
Married	33.6	35.3	39.9	18	25.2	3079
Divorced/separated	29.5	35.5	38.8	19.1	26.8	412
Widowed	26.9	36.8	36.4	16.3	23.5	229
Region						
Kampala	41.4	25.3	45.6	19.6	25.8	1048
Central	43.3	30.8	46.9	18.4	17.4	1032
Eastern	33	62.5	38	29.2	37	1034
Western	21.9	30.4	33.3	7.2	18.9	1039
Northern	29.6	25.2	31.4	14.8	24.8	1045
Education						
No education	30.5	31.5	40.4	18.9	23.1	726
Primary	33.5	34.5	39.9	17.6	24.1	2924
Secondary	35.5	37.2	36.9	17.7	26.9	1381
More than secondary	39.7	36.5	36.5	19.9	27.1	156
Missing	63.6	18.2	18.2	9.1	36.4	11
Wealth quintile						
Lowest	30.3	34.8	33.3	16	24.1	1054
Second	32.5	34.9	37.2	17.1	23.6	1026
Middle	33.9	37.2	41.3	18.5	25.7	1041
Fourth	35.4	34.7	41.6	18.3	25.1	1039
Highest	37.2	32.4	41.7	19.3	25.5	1038
Total	33.8	34.8	39.0	17.8	24.8	5198

Knowledge about sperm washing was reported by only 18% (*n* = 925) while only a quarter (25%, *n* = 1289) knew of PrEP. Knowledge of SCM varied significantly by geographical regions, significantly higher in the Eastern (adj.PR 1.256, CI: 1.191, 1.324), Kampala (adj.PR 1.138, CI: 1.064, 1.218) and central regions (adj.PR 1.136, CI: 1.072, 1.203), Table 4.

**Table 4:** Correlates of knowledge of safer conception methods

Distribution of women, in HIV care, aged 15–49 who reported that they knew any safer conception method (TUI, MSI, PrEP) by selected background characteristics				
Background characteristic	Bivariate		Multivariate	
	PR(95% CI)	*p*-value	PR(95% CI)	*p*-value
Age				
15–19	Ref			
20–24	1.206 (1.024, 1.421)	0.025	1.150(0.977, 1.354)	0.093
25–29	1.254 (1.068, 1.473)	0.006	1.188 (1.011, 1.395)	0.036
30–39	1.237 (1.055, 1.449)	0.009	1.168 (0.996, 1.371)	0.056
40–49	1.235 (1.051, 1.451)	0.010	1.165 (0.990, 1.371)	
Region				
Northern	Ref			
Kampala	1.156 (1.095, 1.220)	0.000	1.138 (1.064, 1.218)	0.000
Central	1.161 (1.099, 1.226)	0.000	1.136 (1.072, 1.203)	0.000
Eastern	1.273 (1.211, 1.339)	0.000	1.256 (1.191, 1.324)	0.000
Western	0.977 (0.918, 1.039)	0.459	0.956 (0.896, 1.019)	0.172
Religion				
Catholic	Ref			
Anglican/Protestant	1.027 (0.987, 1.066)	0.196	1.011 (0.972, 1.051)	0.583
Moslem	1.074 (1.024, 1.127)	0.003	1.016 (0.967, 1.067)	0.526
Pentecostal/Born again/Evangelic	1.028 (0.977, 1.083)	0.288	0.994 (0.944, 1.047)	0.834
SDA	1.020 (0.908, 1.147)	0.736	1.034 (0.919, 1.162)	0.576
Other	1.054 (0.808, 1.373)	0.698	1.108 (0.832, 1.474)	0.482
On ART				
No	Ref			
Yes	1.110 (0.995, 1.239)	0.061		
Duration on ART (Years)				
< 1	Ref			
1	1.019 (0.949, 1.094)	0.593		
2	1.020 (0.954, 1.091)	0.554		
3	1.042 (0.986, 1.101)	0.144		
Currently Married				
No	Ref			
Yes	1.025 (0.992, 1.059)	0.139		
Marital status				
Never married	Ref			
In relationship but not married	1.021 (0.903, 1.154)	0.743		
Married	1.041 (0.923, 1.173)	0.514		
Divorced/Separated	0.995 (0.871, 1.136)	0.941		
Widowed	1.025 (0.890, 1.181)	0.727		
HIV Disclosure to Partner				
No	Ref			
Yes	1.011 (0.966, 1.057)	0.638		
HIV Status of Partner				
Positive	Ref			
Negative	1.031 (0.989, 1.074)	0.145	1.018 (0.977, 1.061)	0.395
DK/DR	0.960 (0.922, 1.000)	0.051	0.957 (0.911, 1.006)	0.083
Education				
No education	Ref			
Primary	1.018 (0.969, 1.069)	0.478		
Secondary	1.033 (0.979, 1.091)	0.233		
More than secondary	1.077 (0.980, 1.184)	0.121		
Missing	1.002 (0.696, 1.443)	0.992		
Wealth Quintiles				
Lowest	Ref			
Second	1.005 (0.952, 1.062)	0.848	1.016 (0.961, 1.073)	0.578
Middle	1.054 (1.001, 1.111)	0.047	1.062 (1.007, 1.119)	0.027
Fourth	1.079 (1.026, 1.136)	0.003	1.089 (1.031, 1.152)	0.003
Highest	1.078 (1.024, 1.134)	0.004	1.055 (0.992, 1.122)	0.090
Level Health Facility				
Hospital	Ref			
Health Center IV	1.001 (0.959, 1.044)	0.975	1.002 (0.958, 1.048)	0.939
Health Center III	1.005 (0.963, 1.048)	0.829	0.994 (0.952, 1.039)	0.801
Health Center II	1.041 (0.979, 1.106)	0.202	0.962 (0.898, 1.031)	0.270
Private Health Unit	1.215 (1.132, 1.305)	0.000	1.142 (1.058, 1.234)	0.001
Others	0.942 (0.728, 1.219)	0.653	0.966 (0.748, 1.248)	0.790
Ownership				
Government/Public	Ref			
Mission/NGO/Private not for profit	1.011 (0.976, 1.047)	0.550		
Private for profit	1.029 (0.902, 1.174)	0.670		

### Correlates of use of safer conception methods

Correlates of use of SCM were computed from 1796 women who were either pregnant at the time of the study or reported a birth in the past 2 years. Ever use of any SCM (TUI, MSI, PrEP and sperm washing) was reported by only 11.6%; lowest among those in the Eastern region (4.6%, n = 18), Table 5. Among those who used SCM (11.6%), majority used TUI (7.2%), followed by PrEP (4%). Only 11 women reported that they had ever used MSI (0.6%) but no woman reported that they had ever used sperm washing (data not shown).

**Table 5:** Use of safer conception methods

Percent distribution of women, in HIV care, aged 15–49 who report that they have ever used any safer conception method (TUI, MSI and PrEP) to reduce HIV transmission by selected background characteristics		
Background characteristic	Percent, %	Number of women
Age		
15–19	14.0	43
20–24	12.4	355
25–29	11.5	529
30–39	23.6	744
40–49	19.5	125
Region		
Kampala	8.2	355
Central	13.8	290
Eastern	4.6	395
Western	13.7	424
Northern	19.3	332
Religion		
Catholic	11.2	723
Anglican / Protestant	14.1	560
Moslem	9.0	256
Pentecostal / Born Again / Evangelical	11.2	215
Others	19.5	42
Marital status		
Never married	5.0	20
In relationship but not married	10.6	396
Married	12.2	1246
Divorced/separated	12.1	99
Widowed	5.7	35
Education		
No education	11.6	224
Primary	12.3	1028
Secondary	10.2	491
More than secondary	14.0	50
Missing	0.0	3
General awareness of Safer Conception		
No	7.0	371
Yes	12.9	1425
Knowledge of any one Safer Conception method		
No	11.8	601
Yes	11.5	1195
Knowledge Categories of Safer Conception		
Knew only one method (low knowledge)	11.8	1394
Knew two methods (moderate knowledge)	13.2	243
Knew 3–4 methods (high knowledge)	8.6	159
Total 15–49	11.6	1796

Table 6 shows that women staying in Kampala (adj.PR 0.489, CI: 0.314, 0.764) and Eastern regions (adj.PR 0.244, CI: 0.147, 0.405) were less likely to use safer conception methods while women who disclosed their HIV status to their partners (adj.PR 2.613, CI: 1.308, 5.221) and those in HIV sero-discordant relationships (adj.PR 1.637, CI: 1.236, 2.168) were more likely to use any SCM. Women who were generally aware of SCM were more likely to use the methods (adj.PR 2.953, CI: (1.968, 4.430) but knowledge of specific methods did not influence use of SCM.

**Table 6:** Correlates of use of safer conception methods

Distribution of women, in HIV care, aged 15–49 who report that they have ever used any safer conception methods (TUI, MSI, PrEP) to reduce HIV transmission by selected background characteristics				
Background characteristic	Bivariate		Multivariate	
	PR(95% CI)	*p*-value	PR(95% CI)	*p*-value
Age				
15–19	Ref			
20–24	0.888 (0.402,1.961)	0.769		
25–29	0.826 (0.379,1.801)	0.631		
30–39	0.809 (0.375, 1.746)	0.589		
40–49	0.803 (0.329, 1.958)	0.629		
Region				
Northern	Ref		Ref	
Kampala	0.424 (0.281,0.640)	0.001	0.489 (0.314,0.764)	0.002
Central	0.716 (0.498,1.028)	0.070	0.818 (0.567,1.179)	0.282
Eastern	0.236 (0.143,0.391)	0.001	0.244 (0.147,0.405)	0.001
Western	0.709 (0.513,0.982)	0.039	0.726 (0.518,1.017)	0.063
Health Facility Level				
Hospital	Ref		Ref	
Health Center IV	1.076 (0.769,1.506)	0.667	1.159 (0.828,1.622)	0.390
Health Center III	1.136 (0.818,1.578)	0.446	1.274 (0.917,1.772)	0.149
Health Center II	0.736 (0.386,1.402)	0.351	1.143 (0.579,2.254)	0.700
Private Health Unit	0.249 (0.036,1.752)	0.163	0.301 (0.041,2.213)	0.238
Others	3.849 (1.579,9.385)	0.003	2.578 (1.001,6.635)	0.050
Religion				
Catholic	Ref			
Anglican / Protestant	1.259 (0.942,1.682)	0.119		
Moslem	0.802 (0.516,1.246)	0.326		
Pentecostal / Born Again Evangelical	0.996 (0.649,1.531)	0.987		
SDA	0.248 (0.035,1.732)	0.160		
Others	1.487 (0.246,9.013)	0.666		
ART				
No	Ref			
Yes	3.775 (0.546,26.110)	0.178		
Duration on ART				
0	Ref			
1	1.325 (0.767,2.288)	0.313		
2	1.479 (0.874,2.501)	0.144		
3+	1.474 (0.929,2.338)	0.100		
Marital status				
Married	Ref			
Not married	1.177 (0.883,1.569)	0.266		
Disclosed HIV status to partner				
No	Ref		Ref	
Yes	3.416 (1.776,6.570)	0.001	2.613 (1.308,5.221)	0.007
Partner’s HIV Status				
Positive	Ref		Ref	
Negative	1.426 (1.076,1.889)	0.013	1.637 (1.236,2.168)	0.001
Don’t know	0.583 (0.392,0.866)	0.008	0.906 (0.595,1.380)	0.646
Partner ART status				
Yes	Ref			
No	1.408 (0.812,2.439)	0.223		
Don’t know	0.685 (0.227,2.063)	0.501		
Education				
No education	Ref			
Primary	1.056 (0.710,1.570)	0.788		
Secondary	0.877 (0.561,1.372)	0.566		
More than secondary	1.206 (0.555,2.622)	0.636		
Number of biological children				
0	Ref			
1	1.086 (0.683,1.726)	0.727		
2	1.047 (0.694,1.581)	0.826		
3	0.685 (0.416,1.128)	0.137		
4+	1.096 (0.756,1.589)	0.629		
Pregnant with an intended pregnancy or intends to have a child in two years				
Yes	Ref			
No	1.102 (0.768, 1.583)	0.597		
General awareness of Safer Conception				
No	Ref		Ref	
Yes	1.838 (1.238,2.727)	0.03	2.953 (1.968,4.430)	< 0.001
Knowledge of any one Safer Conception method				
No	Ref			
Yes	0.977(0.747, 1.279)	0.868		
Knowledge Categories of Safer Conception				
Knew only one method	Ref			
Knew two methods	1.126 (0.798,1.590)	0.497		
Knew three to four methods	0.733 (0.461, 1.168)	0.192		

## Discussion

In this study of knowledge and use of SCM among HIV-infected women receiving HIV care in Uganda, we found that majority of women had heard about SCM and know at least one method, but knowledge on the specific SCM and the actual use of these methods is very low. Only 35 and 39% knew MSI and TUI respectively as methods of safer conception and only 18% knew about sperm washing. SCM methods were ever used by only 11%. These findings show an even lower level of knowledge and use of SCM than what has been shown by other studies [26, 27] and imply a very big missed opportunity for preventing HIV transmission from such a large sexually active HIV positive population (women who had had sex within 12 months).

Our data shows that general knowledge of safer conception increased the likelihood of using the methods (i.e. SCM use was twice as high in those who had general knowledge about safer conception than in those who were not) but knowledge of specific methods did not seem to influence use. This is contrary to what has been documented from prior studies [27] which show that high knowledge is related to increased use of the SCM. The difference in findings may be due to the limited availability of the service although we did not have data to establish service availability. Previous research has shown that safer conception services such as sperm washing remain primarily limited to specialist sites, even in high-income countries [28, 29] and this limits use of the services. The region that had the highest knowledge of SCM mainly knew MSI which has been shown to be among the least used methods [27]. It is also possible that the relatively high knowledge among respondents in some of the regions was due to the effect of pilot safer conception studies that were conducted in TASO Jinja (a big HIV care centre in the Eastern region) and TASO Kampala (a big HIV care centre in Kampala region) that tested the feasibility and acceptability of safer conception counseling among HIV positive clients [19, 26, 27]. However, TASO did not provide a comprehensive service package that could greatly influence use. Indeed, our findings show that even in Kampala and Jinja regions where the pilot studies were conducted, use of SCM remained low, suggesting that while these pilot studies could have succeeded in raising awareness about SCM, they did not influence its use, probably due to lack of access.

Earlier studies done in selected health facilities in Uganda [5, 30], South Africa [20, 23] and Mozambique [22] also revealed that several clients had heard about some of the SCM (especially TUI), but could not provide a comprehensive description of how they are implemented and the clients who reported using methods exhibited partial understanding. Clients with knowledge about safer conception are more likely to open up and initiate child bearing discussions with providers and thus use the service, but need informed providers who will feel more confident talking about issues of conception with HIV positive clients [20]. Knowledge gap among health workers is one of the major reasons why PLHIV do not discuss their fertility desires with providers and hence do not utilize safer conception services [19, 27, 30]. This calls for efforts to improve client and provider education and sensitization about safer conception, while also exploiting the wide coverage of phones and radios that was shown by results of this paper. Addressing this gap will enable effective integration of safer conception into routine family planning services as well as HIV care services.

Higher knowledge and use of SCM was significantly associated with being in an HIV sero-discordant relationship. Discordance is often associated with a dilemma of safer sex practices [7] because of the fear of transmitting HIV infection to partner and child, and this dilemma is likely to yield a search for information on safer conception options and a desire to use them. The increased likelihood of use of SCM among women in HIV sero-discordant relationships is an indication that strengthening safer conception programs with increased focus on clients in HIV sero-discordant relationships may greatly reduce HIV transmission. Increased focus on discordant couples has been shown to increase use of SCM [30]. The fact that 18% of clients who wanted a child or did not use a condom because their partner wanted a child had HIV negative partners further demonstrates a missed opportunity to intercept HIV transmission among these clients, a gap that could be filled if safer conception services were readily available and more clients fully sensitized about them.

Use of SCM was also associated with disclosure of HIV sero-status to one’s sexual partner. Disclosure of HIV status to one’s partner increases partner communication and negotiation on sexual and reproductive health goals and would thus increase chances of using SCM which requires the cooperation of the sexual partner. Involvement of a partner in safer conception counseling ensures that both the man and woman are informed and motivated to accurately employ the appropriate safer conception method [26]. It’s thus important that efforts to increase uptake for safer conception promote disclosure of HIV status and partner involvement. The generally high (83.9%) rate of disclosure among the study population presents an opportunity to introduce safer conception services to as many clients as are in need while aiming to support those that have not disclosed with assisted disclosure services.

Increased knowledge was also associated with being on ART although this did not necessarily influence SCM use. As clients become more stable and healthier on ART, they start to see possibilities of fulfilling their reproductive health needs and probably inquiring about possible alternatives. It is also likely that those who are on ART have stayed in care longer with better adjustment to reproductive health challenges and increased chances of knowing about safer conception options. The limited use of SCM in this group may be explained by the limited availability of the service but also the increasing sensitization in HIV care centres about the need to achieve viral suppression in limiting sexual transmission as well as the increased availability of viral load testing. Clients may find it sufficient to rely on the viral suppression as a strategy to achieve their reproductive desires and find no need to use another method. Although we did not assess use of viral suppression as a safer conception method since guidelines on viral suppression as an SCM had not been rolled out in Uganda, it calls for a need to promote viral suppression both as a prerequisite for use of other SCM but also as a method on its own when one’s viral load becomes sustainably suppressed. Available evidence shows that viral suppression can effectively be used as a safer conception method [31]. Further, only a third of women knew that having an undetectable viral load reduces risk of HIV transmission. This calls for more effort in counseling women and couples on risk prevention, and supporting them to fully understand that HIV viraemia is the strongest risk factor for HIV transmission.

Our study had several limitations. While we acknowledge that women may have several reasons why they do not use safer conception, we did not explore these reasons. Similarly, we did not engage in a more rigorous assessment of comprehensive knowledge of the various aspects of safer conception and SCM which would be key in the implementation of the intervention. We also think that as we were assessing knowledge, respondents may have thought that it is normative to just respond “yes it is true” meaning that ‘they have knowledge’ without giving prior thought to their answers, this could partly explain why we have high levels of knowledge with low SCM use in some regions and low levels of knowledge with high SCM use in other regions. This highlights a need for further research to have a more rigorous assessment of the extent to which knowledge influences use of SCM as well as establish the availability of safer conception services. However the demonstrated very low levels of basic knowledge of SCM and use of safer conception in a large sample of women with high fertility and unprotected sex, is a clear indication of the gap and need to integrate these services both in HIV and family planning services. Previous research has shown that where there is increased availability, of safer conception services, SCM use increases [32].

The other limitation is that this study was conducted among HIV positive women who were in HIV care rather than among all HIV positive women. HIV positive women who are not in HIV care may have different levels of knowledge of safer conception in general and SCM in particular. In addition, we did not include HIV-positive men and HIV negative women at risk of HIV. These are important groups to consider when providing safer conception services and we recommend that further studies and programs include HIV positive women (regardless of their HIV care status), HIV-positive men and HIV negative women at risk of HIV.

Lastly with the growing evidence that sustained viral suppression translates into no viral transmission [31], it would have been of great value to assess how adherent to ART women were (those who were on ART), how this influenced their knowledge and SCM use and how many of them relied on ART and viral suppression as their safer conception strategies. However, we did not assess this since information on viral suppression as a safer conception strategy was not readily available in Uganda at the time of the study.

## Conclusion

Our study found low knowledge and use of safer conception methods among HIV positive women, a population where almost half get pregnant after HIV-positive diagnosis. We found that being in a discordant relationship and having disclosed HIV status to one’s sexual partner were associated with higher levels of knowledge and use of safer conception methods. Collectively, these findings suggest a need for improved sensitization about the safer conception methods, use of innovative ways to integrate safer conception into routine family planning, sexual and reproductive health services and HIV care services at health facilities. Efforts to improve HIV status disclosure as well as support those in HIV-discordant relations who desperately need safer sex solutions will increase availability and uptake of safer conception methods. There is need to fast track the development and roll out of national guidelines on implementation of safer conception and a more concerted regional effort to have safer conception part of basic HIV care.

A French translation of this article has been included as Additional file 1 (see Additional file 1).

A Portuguese translation of the abstract has been included as Additional file 2 (see Additional file 2).

## Additional files

Additional file 1: Translation of this article into French (PDF 405 kb)

Additional file 2: Translation of the abstract of this article into Portuguese (PDF 101 kb)

## Abbreviations

ART: Antiretroviral therapy
CI: Confidence interval
FP: Family planning
HC: Health Centre
IQR: Inter quartile range
MSI: Manual self insemination
PLHIV: People Living with HIV
PR: Prevalence ratio
PrEP: Pre-exposure prophylaxis
SCM: Safer conception methods
SD: Standard deviation
SRH: Sexual and Reproductive Health
SSA: Sub-Saharan Africa
TASO: The AIDS Support Organization
TUI: Timed Unprotected Intercourse
