# Supplementary material for: Knowledge and correlates of use of safer conception methods among HIV-infected women attending HIV care in Uganda
Source: Reprod Health. 2019 May 29;16(Suppl 1):64. doi: 10.1186/s12978-019-0717-6 (PMC6538556; doi:10.1186/s12978-019-0717-6)
Supplement: Supplementary file 1 — Translation of this article into French (PDF 405 kb) [file 12978_2019_717_MOESM1_ESM.pdf]

Connaissances et corrélations de l'utilisation de méthodes de conception plus sûres parmi les femmes infectées par le VIH bénéficiant d'une prise en charge du VIH en Ouganda

Violet Gwokyalya[1*], Jolly Beyeza-Kashesya[2], John Baptist Bwanika[1], Joseph KB Matovu[1], Shaban Mugerwa[3], Jim Arinaitwe[3], Dickson Kasozi[1], Justine Bukenya[1], Rosemary Kindyomunda[4], Glenn J. Wagner[5], Fredrick E Makumbi[1] and Rhoda K. Wanyenze[1]

Affiliations :[1]Makerere University School of Public Health, Kampala, Uganda, [2]Department of Obstetrics and Gynecology, Mulago National Referral Hospital, Kampala, Uganda, [3]Ministry of Health, Kampala, Uganda, [4]UNFPA, Kampala, Uganda, [5]RAND Corporation, Santa Monica, CA, USA.

***Auteur correspondant :** Violet Gwokyalya: vgwokyalya@musph.ac.ug

Adresse électronique des auteurs :
Jolly Beyeza-Kashesya: jbeyeza@gmail.com
John Baptist Bwanika: bwanikajb@yahoo.com
Joseph KB Matovu: jmatovu@musph.ac.ug
Shaban Mugerwa: shabanmugerwa@gmail.com
Jim Arinaitwe: jim_ari@yahoo.co.uk
Dickson Kasozi: dikasozi@gmail.com
Rosemary Kindyomunda: kindyomunda@unfpa.org
Justine Bukenya: jbukenya@musph.ac.ug
Glenn J. Wagner: gwagner@rand.org
Fredrick E Makumbi: fmakumbi@musph.ac.ug
Rhoda K. Wanyenze: rwanyenze@musph.ac.ug

Résumé

Contexte : De nombreuses personnes atteintes du VIH souhaitent avoir des enfants mais les services de planification familiale (PF) se concentrent souvent uniquement sur la contraception. La disponibilité de services de conception plus sûre est encore très faible dans la plupart des pays à faible revenu. Dans cette étude, nous avons évalué les connaissances en matière de méthodes de conception plus sûres (MCS) et leur utilisation parmi les femmes infectées par le VIH et prises en charge pour le VIH en Ouganda pour documenter l'intégration des méthodes de conception plus sûres dans les services de PF existants.

Méthodes : Les données ont été recueillies dans le cadre d'une enquête transversale représentative au niveau national auprès de 5 198 femmes VIH+ âgées de 15 à 49 ans

provenant de 245 centres de prise en charge du VIH en Ouganda. La connaissance et l'utilisation de méthodes de conception plus sûres et les facteurs associés ont été déterminés. Le rapport de prévalence (RP), avec les intervalles de confiance correspondants de 95 %, obtenu à partir d'une régression de Poisson modifiée au moyen de modèles linéaires généralisés, a servi de base à la mesure de la relation. Toutes les analyses ont été menées à l'aide de STATA, version 12.0.

Résultats : La connaissance générale d'une méthode de conception plus sûre était de 74,1 % (3 852/5 198). Cependant, seulement 13,2 % connaissaient 3 à 4 méthodes, 18,9 % seulement 2 méthodes et 42 % connaissaient une seule méthode. La connaissance d'une MCS spécifique était la plus élevée pour les rapports non protégés programmés (RNPP) à 39 % (n=2027) suivie par l'auto-insémination artisanale (AA) à 34,8 % (n=1809) et la prophylaxie pré-exposition (PrEP) à 24,8 % (n=1289). La connaissance des MCS était plus élevée dans l'Est (84,8 %, p<0,001), parmi les femmes en couple sérodiscordant (76,7 %, p<0,017) et sous TAR (74,5 %, p<0,034). Globalement, 1796 (34,6 %) des femmes étaient enceintes ou ont indiqué avoir donné naissance au cours des 2 années précédentes, l'utilisation globale des MCS dans ce groupe étant de 11,6 % (209/1 796). La probabilité d'utilisation des MCS était sensiblement inférieure à Kampala [RP ajusté=0,489 (0,314, 0,764)] ou dans l'Est [RP ajusté=0,244 ; (0,147, 0,405)] par rapport au Nord. Une probabilité plus élevée d'utilisation de MCS était associée à la notification du statut VIH au partenaire [RP ajusté=2,613 (1,308, 5,221)] et à un couple sérodiscordant par rapport à un couple séroconcordant [RP ajusté=1,637 (1,236, 2,168)]. La connaissance préalable de l'une des MCS n'a pas d'influence sur l'utilisation des MCS.

Conclusions : La connaissance et l'utilisation des MCS parmi les femmes VIH+ prises en charge est faible. Les efforts d'amélioration de la notification du statut VIH, l'intégration de la conception plus sûre dans la PF et les services de prise en charge du VIH et les efforts au niveau régional pour augmenter la sensibilisation et l'accès aux méthodes de conception plus sûres peuvent aider à augmenter l'adoption de méthodes de conception plus sûres.

Mots clés : Méthodes de conception plus sûres, connaissances, VIH, Ouganda, santé reproductive

Contexte

L'Ouganda a l'une des populations les plus jeunes et à la croissance la plus rapide du monde ; son taux de fécondité totale est l'un des plus élevés du monde, avec 5,8 enfants par femme [1], avec cependant une prévalence du VIH de 6,2 % [2]. 14 à 73 % des personnes vivant avec le VIH (PVVIH) souhaitent avoir des enfants [3-5]. Environ 40 % des femmes infectées tombent enceintes post-diagnostic VIH [6, 7] et plus de la moitié (57 %) de ces grossesses sont planifiées [6]. Par ailleurs, environ la moitié des couples affectés par le VIH en Ouganda sont sérodiscordants [8] et une part significative (60 %) de nouvelles infections se produit dans ces couples [9, 10]. Les partenaires séronégatifs au VIH dans un couple sérodiscordant, lorsqu'une grossesse survient, ont un risque quasiment deux fois plus important d'être infectés par le VIH par rapport aux couples similaires en l'absence de grossesse [11]. Le traitement antirétroviral (TAR) réduit considérablement le risque de transmission associé à la grossesse, [12], notamment si l'un des deux atteint la suppression virale [13], mais de nombreuses PVVIH ne sont pas encore diagnostiquées, ou le sont mais ne sont pas encore prises en charge, tandis que certaines des personnes sous TAR peuvent ne pas encore avoir atteint la suppression virale pour différentes raisons. Selon le rapport mondial ONUSIDA 2017, seules 60 % des personnes séropositives au VIH sous traitement en Ouganda ont atteint la suppression virale [13]. Par conséquent, l'utilisation de méthodes de conception plus sûres (MCS) telles que l'auto-insémination artisanale (AA), les rapports non protégés programmés (RNPP) et le lavage du sperme (pour ceux qui peuvent se le permettre) reste très importante dans ce contexte pour réduire le risque de transmission par voie sexuelle pendant les essais de procréation [14].

Les directives internationales sur la reproduction, qui recommandaient auparavant d'éviter une grossesse, considèrent depuis dix ans la conception et la parentalité comme des options réalistes et un droit fondamental à la reproduction pour les PVVIH et leur partenaire [15], et encouragent la consultation préconceptionnelle approfondie concernant toutes leurs options de procréation. Depuis 2001, les Centres pour le contrôle et la prévention des maladies américains (US CDC) encouragent également l'information et le soutien aux couples affectés par le VIH qui souhaitent étudier leurs options reproductives [16]. La Société des obstétriciens et gynécologues du Canada [17] et la South African HIV Clinicians Society [18] ont fourni des directives pour une conception plus sûre, avec plusieurs stratégies applicables. Ces directives prévoient : (i) l'encouragement des discussions entre les praticiens et les patients au sujet de la grossesse, (ii) l'identification du désir de procréation des femmes et hommes séropositifs au VIH, en abordant les stratégies de contraception pour les couples qui ne souhaitent pas d'enfants ; et (iii) la prise en charge des personnes et couples affectés par le VIH qui souhaitent une grossesse, en se concentrant sur la prise en charge de l'infection au VIH et des comorbidités avant les essais de procréation, et l'offre de stratégies de conception spécifiques pour les couples positifs au VIH séroconcordants et sérodiscordants. Cependant, ces directives n'ont pas encore été adoptées par les gouvernements dans des contextes à ressources limitées ou intégrées comme norme de soins de routine, et de ce fait l'utilisation des méthodes de conception plus sûres est encore très faible dans la plupart des pays d'Afrique subsaharienne, et en particulier en Ouganda [5, 19, 20]. Les barrières à la mise en œuvre de ces directives comprennent la stigmatisation du patient et des prestataires de soins et le manque de conseil

aux patients concernant la procréation, le manque de connaissances en matière de conception plus sûre parmi les prestataires de soins, et les barrières culturelles [5, 19].

Les PVVIH et leurs prestataires de soins abordent rarement le sujet de la procréation avant la grossesse [21], ce qui représente une opportunité manquée de promouvoir la conception plus sûre et la contraception. Dans la plupart des pays d'Afrique subsaharienne (ASS) [20], dont l'Ouganda [5], la plupart des PVVIH (60-80 %) ne parlent pas de leur désir de procréation avec les prestataires de soins ; les prestataires de soins ont ouvertement découragé leurs patients d'avoir des enfants en raison des risques de transmission et de mortalité (voire les ont réprimandés) [22, 23, 24, 25] tandis que les patients évitent de parler de leurs besoins de procréation avec leurs prestataires de soins en raison de la stigmatisation perçue du prestataire de soins et de la stigmatisation internalisée. Les connaissances approfondies et le conseil en matière de conception plus sûre restent faibles, ce qui a affecté l'utilisation des services de conception plus sûre parmi les patients infectés par le VIH [20]. Une étude réalisée auprès de 48 patients fréquentant un centre de soins spécialisé dans le VIH en Ouganda a montré que 61 % d'entre eux avaient entendu parler d'une ou de plusieurs méthodes de réduction des risques lors de la conception, la méthode la plus connue étant le RNPP (60 %) et la moins connue le lavage du sperme (23 %), mais que seulement 37 % (n=18) des patients étaient en mesure de décrire l'une des méthodes [19]. La plupart des patients considéraient les méthodes de conception plus sûres comme anormales et ont exprimé des inquiétudes, notamment le risque de transmission du VIH, ce qui affecte certainement le recours à ces méthodes. De même, une étude des MCS auprès d'un échantillon de 400 patients ougandais séropositifs au

VIH ayant une relation hétérosexuelle stable et l'intention d'avoir un enfant a montré qu'à peine plus de la moitié d'entre eux savaient que l'AA (53 %) et les RNPP (51 %) réduisaient le risque de transmission à la conception, et que 15 % connaissaient le lavage du sperme et la prophylaxie pré-exposition [26]. Seuls 12 % avaient pratiqué les rapports non protégés programmés lors de leurs essais de procréation, mais aucun n'avait utilisé l'auto-insémination artisanale ou le lavage du sperme [27]. Cependant, la plupart des études précédentes ont été réalisées à petite échelle, et les résultats peuvent ne pas représenter la vaste population VIH prise en charge en Ouganda. Nous avons par conséquent évalué les connaissances et les corrélations de l'utilisation des MCS dans un échantillon, représentatif à l'échelle nationale, de femmes infectées par le VIH dans les cinq régions géographiques de l'Ouganda pour documenter l'intégration de la conception plus sûre dans la PF et les services de santé sexuelle et reproductive (SSR) pour les patients affectés par le VIH.

Méthodes

Conception de l'étude, population et sites

Les données proviennent d'une étude de 5 198 femmes VIH+ faisant partie d'un échantillon représentatif au niveau national de 245 établissements de prise en charge du VIH, privés et publics dans cinq régions géographiques d'Ouganda : Nord, Est, Ouest, Centre et Kampala. Le but de l'étude était d'évaluer la santé sexuelle et reproductive (SSR) et l'intégration des services de planification familiale (PF), les modèles de prestations et le recours aux services de prise en charge du VIH parmi les personnes infectées par le VIH prises en charge. Les établissements de santé ont été sélectionnés selon différents niveaux de prestations de soins de santé en

Ouganda, et comprenaient les hôpitaux et les centres de santé (CSIV, CSIII et CSII) disposant de cliniques de traitement/soins chroniques du VIH. Les centres de santé sont des unités de prestation de services de premier niveau, classés en fonction de la taille de la population desservie, des niveaux d'effectifs et du type de services offerts, le niveau le plus bas étant un centre de santé un (CS I) et le plus haut un centre de santé quatre (CS IV).

Échantillonnage

Un processus d'échantillonnage à deux phases a été utilisé. Au cours de la première phase, un cadre d'échantillonnage comportant une liste d'établissements de prise en charge du VIH a permis de sélectionner un nombre identique d'établissements dans chaque région. La deuxième phase de l'échantillonnage a consisté en la sélection des participants à l'étude au niveau des établissements. Toutes les femmes VIH+ âgées de 15 à 49 ans qui se présentaient dans les centres sélectionnés pour des soins VIH le jour des entretiens étaient enregistrées sur les registres de présence quotidienne de leur service. Un échantillonnage systématique a ensuite été mené pour sélectionner aléatoirement le nombre requis de femmes éligibles à partir des listes de présence quotidienne. Après l'échantillonnage, un outil de sélection succinct a permis d'évaluer l'éligibilité sur la base de l'âge (seules les femmes âgées de 15 à 49 étaient admissibles pour cette étude) et de l'activité sexuelle (seules les femmes ayant eu au moins un rapport sexuel au cours des 12 derniers mois étaient admissibles). Les participantes éligibles ont fourni leur consentement écrit avant qu'il ne soit procédé à l'entretien complet.

Taille de l'échantillon

Le calcul de la taille de l'échantillon reposait sur le besoin non satisfait de PF en tant que résultat principal de l'étude, en partant de l'hypothèse d'un besoin non satisfait de PF de 30 % parmi les femmes VIH+ prises en charge, une marge d'erreur de 3,6 %, un taux d'erreur de type I de 5 %, un effet de plan d'1,5 et un taux de non-réponse de 10 %. Sur la base de ces hypothèses, nous avons obtenu une taille d'échantillon de 5 185 dans les cinq régions géographiques (c'est-à-dire 1 037 répondantes par région). 20 participantes environ ont été sélectionnées dans chaque établissement, à l'exception des établissements de plus grande taille de la région de Kampala, avec >5 000 patients pris en charge pour le VIH, où 30 à 50 patientes ont été sélectionnées aléatoirement dans chaque établissement. Kampala a moins d'établissements de soins du VIH par rapport aux autres régions, mais leur volume de patients est plus important. Dans l'ensemble, les participantes ont été sélectionnées à partir de 52 établissements dans chaque région, à l'exception de Kampala, où les participantes ont été sélectionnées dans 37 établissements agréés pour le TAR.

Méthodes et procédures de collecte des données

Les données concernant les connaissances générales des méthodes de conception plus sûres, à savoir RNPP, AA, PrEP pour les partenaires séronégatifs au VIH et le lavage du sperme ont été collectées à l'aide d'un questionnaire structuré administré par un enquêteur. Les femmes ont répondu à une série de questions leur demandant si elles connaissaient les MCS, si elles avaient utilisé une de ces MCS séparément ou une combinaison de ces MCS pour les femmes enceintes ou ayant indiqué avoir donné naissance au cours des 2 années précédentes. D'autres questions concernant la conception plus sûre, comprenant des questions sur les stratégies de réduction

du risque de transmission du VIH lors de la planification de la conception, par exemple la mise en place précoce du TAR pour les personnes infectées par le VIH, et le fait d'avoir une charge virale indétectable. Les femmes ont également répondu à des questions concernant leurs antécédents génésiques ; leurs enfants nés vivants, leur désir d'avoir un ou plusieurs enfants et la planification de la grossesse suivante. Les données ont été collectées de septembre à novembre 2016 par une équipe de 30 enquêteurs qualifiés et expérimentés. Les outils ont été pré-testés pour vérifier l'adéquation de différents aspects des questionnaires tels que la traduction, les procédures de saut et de filtre des questions, et des modifications ont été apportées avant la collecte des données à proprement parler. Tous les outils de collecte des données ont été traduits dans les langues courantes des régions sélectionnées.

Mesures

La variable de résultat primaire était l'indication de la connaissance de méthodes de conception plus sûres, ou l'utilisation d'une MCS. La connaissance des méthodes de conception plus sûres a été évaluée à l'aide de plusieurs affirmations concernant la connaissance générale de l'existence de ces méthodes et des affirmations spécifiques à chaque méthode, qui étaient lues aux répondantes, avec des réponses codées telles que « Vrai », « Faux », ou « Je ne sais pas ». Les affirmations de connaissance générale de l'existence visaient à étudier les connaissances des répondantes relatives au concept de « conception plus sûre » en général (par exemple *« Il existe des manières de rendre plus sûre la conception avec un partenaire négatif au VIH »)* tandis que des questions spécifiques aux méthodes étudiaient les connaissances spécifiques des répondantes concernant les différentes MCS (par exemple le RNPP – « *Avoir des rapports non protégés pendant les jours du mois où une femme est plus fertile aidera à limiter le risque*

de transmission du VIH à un partenaire non infecté »). Les répondantes qui répondaient « Vrai » à l'une des affirmations générales ou spécifiques à une méthode étaient réputées connaître la conception plus sûre en général et les MCS en particulier. Les répondantes qui connaissaient les MCS étaient regroupées en répondantes connaissant : a) 3 à 4 méthodes (indiquées comme ayant un « niveau de connaissance élevé » des MCS) ; b) seulement deux méthodes (indiquées comme ayant un « niveau de connaissance moyen ») ou c) une seule méthode (indiquées comme ayant un « faible niveau de connaissance »). L'utilisation des méthodes de conception plus sûres a été déterminée auprès de femmes ayant indiqué avoir donné naissance au cours des 2 années précédentes ou étant enceintes. Une femme était classée comme utilisatrice d'une MCS si elle indiquait utiliser une des MCS (RNPP, AA, PrEP pour le partenaire non infecté pour celles qui étaient en couple sérodiscordant, et lavage du sperme) pour réduire le risque de transmission du VIH lors des essais de procréation avec leur partenaire

Gestion des données et analyse

Nous avons mené des analyses descriptives pour décrire les caractéristiques sociodémographiques des femmes de l'échantillon, le diagnostic de VIH et le statut de traitement, les caractéristiques du partenaire, dont son statut VIH, la connaissance et l'utilisation de MCS. Les relations entre la connaissance ou l'utilisation de MCS et les caractéristiques de la femme ont été mesurées au moyen du rapport de prévalence (RP). Le RP a été obtenu au moyen d'un modèle de régression de Poisson « modifié » par un modèle linéaire généralisé avec family=Poisson et link=log et des erreurs standard robustes. Toutes les analyses ont été réalisées au moyen de Stata version 12. Dans l'analyse bivariée utilisée pour

déterminer les facteurs indépendants associés aux résultats, les modèles comprenaient les covariables suivantes : âge, région, religion, niveau et propriétaire de l'établissement de santé, niveau d'éducation, quintile de richesse, statut marital, statut TAR de la patiente et durée sous TAR, notification de la séropositivité au partenaire, statut VIH du partenaire, statut TAR du partenaire s'il est lui aussi séropositif au VIH, et nombre d'enfants biologiques. Des variables considérées comme significatives à partir d'un seuil de signification de 5 % et des variables importantes provenant d'études précédentes ont été incluses dans l'analyse multivariée.

Résultats

Caractéristiques des participantes

Le tableau 1 indique les caractéristiques des 5 198 femmes prises en compte dans cette analyse. L'âge moyen (écart standard) était de 32,5 (7,2) ans, et 85,6 % étaient soit mariées (59,2 %, n=3 079) soit en couple (26,4 %, n=1 371). La mise sous TAR était quasiment universelle (96,6 % ; n=5 022), avec une période médiane (EI) de 3 (2, 6) ans, 57,4 %(n=2 985) étaient en couple séroconcordant et 18,4 % (n=954) en couple sérodiscordant, le reste ne connaissait pas le statut VIH de leur partenaire. Près d'un quart (23 %, n=1 168) des femmes ayant participé à l'étude étaient enceintes (5,6 %) ou voulaient un enfant (17,5 %) et 28 % (n=1 422) n'avaient pas utilisé de préservatif lors de leur dernier rapport sexuel parce que leur partenaire voulait un enfant. Parmi les femmes souhaitant procréer, 18 % (n=122) avaient un partenaire négatif au VIH. La notification du statut séropositif au VIH à un partenaire sexuel était de 84,5 % (n=4 362) ; sensiblement supérieure (p<0,001) dans les couples séroconcordants

(97,9 %, ; n=2 922) par rapport aux couples sérodiscordants (89 % ; n=849) et aux couples dont le statut VIH du partenaire n'était pas connu (46,9 % ; n=590).

Connaissance des méthodes de conception plus sûre et modes de réduction du risque de transmission du VIH

Globalement, 80,2 % (n=4 262) avaient une connaissance générale des méthodes de conception plus sûres et 74,1 % (n=3 852) connaissaient au moins une MCS. Parmi celles qui connaissaient au moins une MCS, 42 % (n=2 183) connaissaient une seule méthode (faible niveau de connaissance), 18,9 % (n=986) connaissaient seulement deux méthodes (niveau de connaissance moyen) et 13,2 % (n=685) connaissaient trois ou quatre MCS (niveau de connaissance élevé). Le Tableau 2 montre que la connaissance d'une MCS était plus élevée dans la région de l'Est (84,8 %, n=877, p<0,001), parmi les femmes en couple sérodiscordant (76,7 %, n=731, p<0,017), parmi les femmes ayant une situation économique plus favorable (p<0,003) et parmi les femmes sous TAR (74,5 %, n=3741, p<0,034). La connaissance d'une MCS spécifique était plus faible pour l'AA (35 %, n=1 809) par rapport au RNPP (39,0 %, n=2 027), Tableau 3. La connaissance du RNPP était plus élevée dans les régions du Centre (47 %) et de Kampala (46 %), tandis que celle de l'AA était plus élevée dans la région de l'Est, ou 63 % des femmes savaient qu'un homme pouvait éjaculer dans un préservatif ou dans un récipient et introduire manuellement le sperme dans le vagin d'une femme.

Seulement 18 % (n=925) indiquaient connaître le lavage du sperme tandis qu'un quart (25 %, n=1289) connaissait la PrEP. La connaissance des MCS variait sensiblement d'une région à

l'autre, et était nettement plus élevée dans la région de l'Est (RP ajusté 1,256, IC : 1,191, 1,324), de Kampala (RP ajusté 1,138, IC : 1,064, 1,218) et du Centre (RP ajusté 1,136, IC : 1,072, 1,203), Tableau 4.

Corrélations de l'utilisation de méthodes de conception plus sûres

Les corrélations de l'utilisation des MCS ont été calculées à partir de 1 796 femmes enceintes au moment de l'étude ou ayant indiqué avoir donné naissance au cours des 2 années précédentes. L'utilisation d'une MCS (RNPP, AA, PrEP et lavage du sperme) a été indiquée par seulement 11,6 % des répondantes, et était la plus faible dans la région de l'Est (4,6 %, n=18), Tableau 5. Parmi les femmes ayant utilisé une MCS (11,6 %), la majorité avait utilisé le RNPP (7,2 %), suivi par la PrEP (4 %). Seules 11 femmes ont indiqué avoir eu recours à l'AA (0,6 %) mais aucune femme n'a indiqué avoir eu recours au lavage du sperme (donnée non représentée).

Le Tableau 6 indique que les femmes résidant dans la région de Kampala (RP ajusté 0,489, IC : 0,314, 0,764) et de l'Est (RP ajusté 0,244, IC : 0,147, 0,405) étaient moins susceptibles d'utiliser des méthodes de conception plus sûres tandis que les femmes qui avaient notifié leur statut VIH à leur partenaire (RP ajusté 2,613, IC : 1,308, 5,221) et les femmes en couple sérodiscordant (RP ajusté 1,637, IC : 1,236, 2,168) étaient plus susceptibles d'utiliser une MCS. Les femmes ayant une connaissance générale de l'existence des MCS étaient plus susceptibles de les utiliser (RP ajusté 2,953, IC : (1,968, 4,430) mais la connaissance d'une méthode spécifique n'avait pas d'influence sur l'utilisation des MCS.

Discussion

Dans cette étude de la connaissance et de l'utilisation des MCS parmi les femmes infectées par le VIH et prises en charge pour le VIH en Ouganda, nous avons constaté qu'une majorité de femmes avait entendu parler des MCS et connaissait au moins une méthode, mais que la connaissance de MCS spécifiques et leur utilisation effective étaient très faibles. Seules 35 % des femmes connaissaient l'AA et 39 % les RNPP comme méthodes de conception plus sûre, et seules 18 % avaient entendu parler du lavage du sperme. Les MCS ont été utilisées par 11 % seulement des répondantes. Ces résultats montrent un niveau encore inférieur de connaissances et d'utilisation des MCS par rapport au niveau rencontré dans d'autres études [26, 27] et représente une très grande occasion manquée dans la prévention de la transmission du VIH par une population séropositive au VIH si nombreuse et sexuellement active (femmes ayant eu des rapports sexuels au cours des 12 derniers mois).

Nos données indiquent que la connaissance générale de la conception plus sûre a augmenté la probabilité d'utilisation des méthodes (l'utilisation des MCS était deux fois plus élevée chez les femmes ayant une connaissance générale de la conception plus sûre que chez les femmes qui n'en avaient pas), mais que la connaissance de méthodes spécifiques ne semblait pas en influencer l'utilisation. Ceci est contraire au phénomène documenté dans des études précédentes [27], qui indiquent qu'un niveau de connaissance élevé est lié à une utilisation accrue des MCS. La différence de résultats peut être due à la disponibilité limitée des services, bien que nous n'ayons pas de données nous permettant d'établir la disponibilité des services. Des recherches précédentes ont montré que les services de conception plus sûre tels que le

lavage du sperme restent principalement limités à des établissements spécialisés, même dans les pays à haut revenu [28, 29], et que ceci limite l'utilisation de ces services. La région présentant le niveau de connaissance le plus élevé des MCS connaissait principalement l'AA, qui est l'une des méthodes les moins utilisées [27]. Il est également possible que le niveau de connaissance relativement élevé parmi les répondantes dans certaines régions soit dû à l'impact d'études de conception plus sûre pilotes menées à TASO Jinja (grand centre de prise en charge du VIH dans la région de l'Est) et à TASO Kampala (grand centre de prise en charge du VIH dans la région de Kampala) qui ont testé la faisabilité et l'acceptabilité du conseil en conception plus sûre parmi les patients positifs au VIH [19, 26, 27]. Cependant, TASO n'a pas fourni d'offre de services complète en mesure d'influencer significativement l'utilisation. En effet, nos résultats montrent que, même dans les régions de Kampala et de Jinja, où les études pilotes ont été menées, l'utilisation des MCS reste faible, ce qui laisse à penser que, bien que ces études pilotes aient réussi à sensibiliser les patientes aux MCS, elles n'ont pas influencé leur utilisation, probablement en raison d'une difficulté d'accès.

Des études précédentes réalisées auprès d'établissements de santé sélectionnés en Ouganda [5, 30], en Afrique du Sud [20, 23] et au Mozambique [22] ont elles aussi révélé que plusieurs patientes avaient entendu parlé de certaines MCS (notamment les RNPP), mais ne pouvaient pas fournir une description complète de leur application, et que les patientes ayant indiqué utiliser les méthodes ne les comprenaient qu'en partie. Les patientes connaissant la conception plus sûre sont plus susceptibles d'être ouvertes à des discussions sur la procréation et de les engager avec les prestataires de soins, et ainsi de recourir aux services, mais elles ont besoin de prestataires de soins informés qui se sentent plus confiants pour aborder le sujet de la

conception avec des patients séropositifs au VIH [20]. L'écart de connaissances parmi les professionnels de la santé est l'une des raisons principales pour lesquelles les PVVIH n'abordent pas leur désir d'enfant avec les prestataires de soins et par conséquent n'utilisent pas les services de conception plus sûre [19, 27, 30]. Ceci indique la nécessité d'efforts pour améliorer l'éducation des patientes et des prestataires de soins et la sensibilisation à la conception plus sûre, tout en exploitant la vaste couverture offerte par les téléphones et les radios montrée dans les résultats de cet article. S'attaquer à cet écart permettra l'intégration efficace de la conception plus sûre dans les services de planification familiale de routine ainsi que dans les services de prise en charge du VIH.

Un niveau de connaissance et d'utilisation plus élevé des MCS a été nettement associé aux couples sérodiscordants. La discordance est souvent associée au dilemme des pratiques sexuelles plus sûres [7] en raison de la peur de transmettre l'infection au VIH au partenaire et à l'enfant, et ce dilemme est susceptible de susciter une recherche d'informations sur les options de conception plus sûre et le souhait de les appliquer. Cette probabilité accrue d'utiliser les MCS parmi les femmes en couple sérodiscordant est une indication que renforcer les programmes de conception plus sûre en se concentrant davantage sur les patients en couple sérodiscordant peut considérablement réduire la transmission du VIH. Il a été démontré que l'attention accrue aux couples discordants augmente l'utilisation des MCS [30]. Le fait que 18 % des patientes qui souhaitent avoir un enfant ou qui n'ont pas utilisé de préservatif car leur partenaire souhaite un enfant aient un partenaire séronégatif au VIH indique une occasion manquée de lutter contre la transmission du VIH parmi ces patients, lacune qui pourrait être

comblée si des services de conception plus sûre étaient déjà disponibles et si plus de patients y étaient sensibilisés.

L'utilisation des MCS était également associée à la notification du statut séropositif au VIH au partenaire sexuel. La notification du statut VIH au partenaire augmente la communication entre les partenaires et la négociation des objectifs de santé sexuelle et reproductive et augmenterait ainsi les probabilités d'utiliser des MCS qui nécessitent la coopération du partenaire sexuel. L'implication d'un partenaire dans le conseil pour une conception plus sûre permet de s'assurer que l'homme et la femme sont informés et encouragés à utiliser scrupuleusement la méthode de conception plus sûre appropriée [26]. Ainsi est-il important que les efforts pour augmenter le recours à la conception plus sûre encouragent la notification du statut VIH et l'implication du partenaire. Le taux de notification généralement élevé (83,9 %) parmi la population étudiée représente une opportunité d'introduire des services de conception plus sûre auprès patients en ayant besoin tout en visant à apporter un soutien à ceux qui n'ont pas informé leur partenaire de leur statut à l'aide de services d'assistance à la notification.

L'accroissement des connaissances a également été associé au fait d'être sous TAR, même si ceci n'influençait pas forcément l'utilisation d'une MCS. À mesure que les patients se stabilisent et retrouvent la santé sous TAR, ils commencent à entrevoir des possibilités de répondre à leur besoin de procréation et sont susceptibles de s'informer sur les alternatives possibles. Il est également probable que les personnes sous TAR soient prises en charge depuis plus longtemps, avec un meilleur ajustement aux défis de la santé reproductive, et des possibilités accrues de

connaître les méthodes de conception plus sûres. L'utilisation limitée des MCS dans ce groupe peut s'expliquer par la disponibilité limitée des services, mais également par la sensibilisation accrue dans les centres de prise en charge du VIH au besoin d'atteindre la suppression virale en limitant la transmission sexuelle et par la disponibilité accrue du test de la charge virale. Les patients peuvent trouver suffisant de se fier à la suppression virale comme stratégie de réponse à leur désir de procréation et ne pas trouver nécessaire d'utiliser une autre méthode. Bien que nous n'ayons pas évalué la suppression virale comme méthode de conception plus sûre étant donné que les directives concernant la suppression virale comme MCS n'avaient pas été déployées en Ouganda, ceci plaide pour un besoin de promouvoir la suppression virale autant comme prérequis à l'utilisation d'autres MCS, mais également comme méthode en tant que telle quand la charge virale d'une personne est supprimée durablement. Les données disponibles montrent que la suppression virale peut être utilisée efficacement comme méthode de conception plus sûre [31]. Par ailleurs, seul un tiers des femmes savaient qu'une charge virale indétectable réduit le risque de transmission du VIH. Ceci plaide pour des efforts supplémentaires dans les conseils aux femmes et aux couples sur la prévention des risques, et pour leur faire comprendre pleinement que la virémie VIH est le facteur de risque le plus important pour la transmission du VIH.

Notre étude présente plusieurs limites. Même si nous constatons que les femmes peuvent avoir plusieurs raisons de ne pas utiliser la conception plus sûre, nous n'avons pas examiné ces raisons. De même, nous ne nous sommes pas lancés dans une évaluation plus rigoureuse des connaissances approfondies concernant les différents aspects de la conception plus sûre et des

MCS qui joueraient un rôle clé dans la mise en œuvre de l'intervention. Nous pensons également que, lorsque nous avons évalué les connaissances, les répondantes peuvent avoir pensé qu'il était normatif de répondre « oui c'est vrai » pour dire qu'elles « avaient des connaissances », sans penser à leur réponse, ce qui pourrait expliquer en partie pourquoi nous observons un niveau de connaissances élevé et une faible utilisation des MCS dans certaines régions, et un bas niveau de connaissances avec une utilisation importante des MCS dans d'autres. Ceci met en évidence un besoin de poursuivre la recherche pour obtenir une évaluation plus rigoureuse de la portée de l'influence des connaissances sur l'utilisation des MCS et établir la disponibilité des services de conception plus sûre. Nous avons cependant démontré qu'un très bas niveau de connaissances de base des MCS et d'utilisation de la conception plus sûre auprès d'un vaste échantillon de femmes à fécondité élevée ayant des rapports sexuels non protégés est une indication claire de l'écart à combler et du besoin d'intégrer ces services dans les services de prise en charge du VIH et de planification familiale. Des études précédentes ont indiqué que, quand la disponibilité des services de conception plus sûre augmente, l'utilisation des MCS augmente lui aussi [32].

L'autre limite est que cette étude a été menée auprès de femmes séropositives au VIH prises en charge pour le VIH, et non auprès de toutes les femmes séropositives au VIH. Les femmes séropositives au VIH qui ne sont pas prises en charge peuvent avoir un niveau de connaissances différent de la conception plus sûre en général et des MCS en particulier. Par ailleurs, nous n'avons pas inclus les hommes séropositifs au VIH et les femmes séronégatives à risque de VIH. Il est important de prendre ces groupes en compte lors de la fourniture de services de

conception plus sûre, et nous recommandons que d'autres études et programmes incluent les femmes séropositives au VIH (quel que soit leur statut de prise en charge), les hommes séropositifs au VIH et les femmes séronégatives à risque de VIH.

Enfin, alors que les preuves qu'une suppression virale durable se traduit par une absence de transmission du virus augmentent [31], il aurait été très utile d'évaluer l'adhésion au TAR des femmes (sous TAR), la manière dont ceci influence leur connaissance et leur utilisation des MCS et le nombre d'entre elles qui s'appuyaient sur le TAR et la suppression virale comme stratégie de conception plus sûre. Cependant, nous n'avons pas évalué ces aspects car les informations sur la suppression virale comme stratégie de conception plus sûre n'étaient pas facilement disponibles en Ouganda au moment de l'étude.

Conclusion

Notre étude a mis en évidence un bas niveau de connaissances et d'utilisation des méthodes de conception plus sûres parmi les femmes séropositives au VIH, population dont au moins une moitié tombe enceinte après un diagnostic de VIH. Nous avons mis en évidence que le fait d'être en couple sérodiscordant et d'avoir notifié son statut VIH au partenaire sexuel était associé à une niveau de connaissance et d'utilisation de méthodes de conception plus sûres plus élevé. Dans l'ensemble, ces résultats suggèrent un besoin d'améliorer la sensibilisation aux méthodes de conception plus sûres, d'utiliser de nouvelles manières d'intégrer la conception plus sûre de manière habituelle dans la planification familiale, les services de santé sexuelle et reproductive et les services de prise en charge du VIH dans les établissements de santé. Les

efforts d'amélioration de la notification du statut VIH ainsi que le soutien aux personnes en couple sérodiscordant à la recherche désespérée de solutions pour des rapports sexuels plus sûrs augmentera la disponibilité envers les méthodes de conception plus sûres et le recours à ces dernières. Il est nécessaire d'accélérer le développement et le déploiement de directives nationales concernant l'application de la conception plus sûre et d'une plus grande concertation des efforts régionaux pour intégrer la conception plus sûre dans la prise en charge de base du VIH.

Liste des abréviations

TAR	Traitement antirétroviral
IC	Intervalle de confiance
PF	planification familiale
CS	Centre de santé
EI	Écart interquartile
AA	Auto-insémination artisanale
PVVIH	personnes vivant avec le VIH
RP	Rapport de prévalence
PrEP	Prophylaxie préexposition
MCS	Méthodes de conception plus sûres
ET	Écart type
SSR	Santé sexuelle et reproductive
ASS	Afrique Subsaharienne
TASO	ONG « The AIDS Support Organization »
RNPP	Rapports non protégés programmés

Concernant ce supplément

Cet article a été publié dans l'ouvrage *Reproductive Health*, Volume 16 Supplement 1, 2019: Effective Integration of Sexual Reproductive Health and HIV Prevention, Treatment, and Care Services across sub-Saharan Africa: Where is the evidence for program implementation?
Le supplément a été publié dans le cadre d'une collaboration entre *Reproductive Health* et *BMC Public Health.* L'intégralité du contenu, avec les versions en français, en portugais et en anglais, est disponible en ligne :
https://bmcpublichealth.biomedcentral.com/articles/supplements/volume-19-supplement-1
et
https://reproductive-health-journal.biomedcentral.com/articles/supplements/volume-16-supplement-1

Déclarations

Approbation éthique et accord de participation

L'approbation éthique de cette étude a été conférée par le Comité « Higher Degrees, Research and Ethics » de la School of Public Health de l'Université Makerere et par le Conseil national de la science de la technologie de l'Ouganda. Les directeurs de district et d'établissement ont également autorisé l'étude. Les participantes étaient anonymes et ont fourni leur consentement éclairé par écrit, qui était séparé du questionnaire. Les participantes ont reçu 5 000 shillings ougandais (1,5 US$) en compensation de leur temps, sur autorisation de l'IRB. Les adolescentes sexuellement actives (15-17 ans) ont été traitées comme des mineures émancipées, avec dispense du consentement des parents/du responsable légal, sur la base des directives nationales pour les recherches [33].

Accord de publication

Non applicable

Disponibilité des données et matériels

Le ou les fichiers de données étayant les conclusions de cet article sont disponibles sur demande et sur autorisation de l'UNFPA, du ministère de la Santé ougandais et de l'Institutional Review Board de l'Université Makerere.

Conflits d'intérêts

Les auteurs déclarent ne pas avoir de conflits d'intérêts.

Financement

Cette étude a été financée par le ministère de la Santé ougandais, par une bourse du Fonds Mondial et de l'UNFPA. La publication de cet article a été financée par l'UNFPA. Les financeurs n'ont joué aucun rôle dans la conception de l'étude, la collecte des données et l'analyse, la décision de publier et la préparation du manuscrit.

Le supplément de la revue est rendu possible grâce au soutien généreux du peuple américain via la United States Agency for International Development (USAID) en partenariat avec le Fonds des Nations unies pour la population (FNUAP) et le Programme commun des Nations Unies sur le VIH/SIDA (ONUSIDA).

Les opinions exprimées dans la présente publication sont celles des auteurs et ne reflètent pas nécessairement les politiques officielles de l'USAID, du FNUAP ou de l'ONUSIDA, la mention des dénominations de ministères ou d'organismes n'implique pas non plus l'aval du gouvernement américain, du FNUAP ou de l'ONUSIDA.

Contributions des auteurs

VG, JBK, JBB, JKBM, SM, JA, JB, DK, RK, FM et RKW ont lancé le sujet, développé la proposition et coordonné la collecte des données. JBB et FM ont analysé les données. VG a rédigé la première version du manuscrit. Tous les auteurs ont participé à l'interprétation des données, ont relu et approuvé le manuscrit final.

Remerciements

Nous remercions le ministère de la Santé ougandais et la direction de la School of Public Hearth de l'université Makerere, les directeurs de districts et d'établissements ainsi que les patientes qui ont participé à l'étude.

RÉFÉRENCES

1. Uganda Demographic and Health Survey 2016. https://www.ubos.org/onlinefiles/uploads/ubos/pdf%20documents/Uganda_DHS_2016_KIR.pdf (2017). Accessed 1 Aug 2018

2. Uganda Population-based HIV impact assessment UPHIA 2016-2017. https://www.scribd.com/document/356607702/UPHIA-Uganda-Factsheet (2017). Accessed 2 Aug 2018.

3. Beyeza-Kashesya J, Ekstrom AM, Kaharuza F, Mirembe F, Neema S, Kulane A. My partner wants a child: a cross-sectional study of the determinants of the desire for children among mutually disclosed sero-discordant couples receiving care in Uganda. BMC Public Health. 2010;10:247.

4. Kakaire O, Osinde M, Kaye DK. Factors that predict fertility desires for people living with HIV infection at a support and treatment centre in Kabale, Uganda. Reprod Health. 2010; 7: 27

5. Wagner G, Linnemayr S, Kityo C, Mugyenyi P. Factors Associated with Intention to Conceive and its Communication to Providers Among HIV Clients in Uganda. Matern Child Health J. 2012; 16(2):510-8

6. Wanyenze RK, et al. Uptake of Family Planning methods and unplanned pregnancies among HIV infected individuals: A cross-sectional survey among clients at HIV clinics in Uganda. J Int AIDS Soc. 2011; 14: 35

7. Beyeza-Kashesya J, Kaharuza F, Mirembe F, et al. The dilemma of safe sex and having children: challenges facing HIV sero-discordant couples in Uganda. African Health Services 2009; 9:2-12.

8. Uganda Ministry of Health. Uganda AIDS Indicator Survey 2011. https://dhsprogram.com/pubs/pdf/AIS10/AIS10.pdf. Accessed 12 Nov 2017.

9. Allen S, Meinzen-Derr J, Kautzman M, Zulu I, Trask S, Fideli U, et al. Sexual behavior of HIV discordant couples after HIV counseling and testing. AIDS. 2003; 17(5):733-40

10. Tadesse M. Assessment of HIV discordance and associated risk factors among couples receiving HIV test in Dilla, Ethiopia. BMC Research Notes. 2014; 7:893.

11. S.G. Brubaker, E. A. Bukusi, J. Odoyo et al., "Pregnancy and HIV transmission among HIV-discordant couples in a clinical trial in Kisumu, Kenya," HIV Med. 2011; 12(5):316-21.

12. UNICEF. Count Down to Zero: Elimination of New HIV Infections Among Children by 2015 And Keeping Their Mothers Al*ive (Uganda).* https://data.unicef.org/wp-content/uploads/country_profiles/Uganda/PMTCT_UGA.pdf (2012). Accessed 27 Jul 2018.

13. Joint United Nations Programme on HIV/AIDS (UNAIDS) special analysis, 2017. Ending AIDS Progress towards the 90–90–90 targets. http://www.unaids.org/sites/default/files/media_asset/Global_AIDS_update_2017_en.pdf (2017). Accessed 1 Aug 2018

14. Mmeje O. Call for integration of safer conception and reproductive health services into HIV care and treatment programs for HIV serodiscordant couples desiring conception. http://integrationforimpact.org/wp-content/uploads/2012/10/A_18_Mmeje_Calls-for-Integration-of-Safer-Conception.pdf (2012). Accessed 30 Jan 2017

15. Fakoya A, Lamba H, Mackie N, et al. British HIV Association, BASHH and FSRH guidelines for the management of the sexual and reproductive health of people living with HIV infection 2008. HIV Med. 2008;9:681-720

16. Centers for Disease Control and Prevention. Revised guidelines for HIV counseling, testing, and referral. MMWR Recomm Rep. 2001;50(RR-19):1-57

17. Loutfy MR, Margolese S, Money DM, Gysler M, Hamilton S, Yudin MH. Canadian HIV Pregnancy Planning Guidelines: No. 354, January 2018. Int J Gynaecol Obstet. 2018; 40(1): 94-114

18. Bekker L-G, Black V, Myer L, Rees H, Cooper D, Mall S, Mnyami C, Conradie F, Mahabeer I, Gilbert L, Schwartz S. Guideline on safer conception in fertile HIV-infected individuals and couples. Southern African Journal of HIV Medicine. 2011; 12(2): 31-44.

19. Finocchario KS, Wanyenze R, Mindry D, Beyeza KJ, Goggin K, Nabiryo C, Wagner G. "I may not say we really have a method, it is gambling work": Knowledge and acceptability of safer conception methods among providers and HIV clients in Uganda. Health Care Women Int. 2014; 35(0): 896–917.

20. Schwartz SR, Mehta SH, Taha TE, Rees HV, Venter F, Black V. High pregnancy intentions and missed opportunities for patient-provider communication about fertility in a South African cohort of HIV-positive women on antiretroviral therapy. AIDS Behav. 2012;16:69–78.

21. Beyeza-Kashesya J, Wanyenze RK, Goggin K, Finocchario-Kessler S, Woldetsadik MA, Mindry D, Birungi J, Wagner GJ. Stigma gets in my way: Factors affecting client-provider communication regarding childbearing among people living with HIV in Uganda. PLoS One. 2018;13(2):e0192902.

22. Agadjanian V, Hayford SR. PMTCT, HAART, and childbearing in Mozambique: an institutional perspective. AIDS Behav 2009; 13 Suppl 1:103-112.

23. Cooper D, Moodley J, Zweigenthal V, et al. Fertility intentions and reproductive health care needs of people living with HIV in Cape Town, South Africa: implications for integrating reproductive health and HIV care services. AIDS Behav. 2009;13 Suppl 1:38-46.

24. Goggin K, Mindry D, Beyeza-Kashesya J, et al. “Our hands are tied up”: Current state of safer conception services suggests the need for an integrated care model. Health Care Women Int. 2014; 35(0): 990–1009

25. Moodley J, Cooper D, Mantell JE, Stern E. Health care provider perspectives on pregnancy and parenting in HIV-positive individuals in South Africa. BMC Health Serv Res. 2014; 14: 384.

26. Wagner G, Woldetsadik MA, Beyeza-Kashesya J, Goggi K, Mindry D, Finocchario-Kessler S, Khanakwa S, and Wanyenze RK. Multi-level Correlates of Safer Conception Methods Awareness and Attitudes Among Ugandan HIV Clients with Fertility Intentions. Afr J Reprod Health. 2016; 20(1): 40–51.

27. Wagner G, Goggi K, Mindry D, Beyeza-Kashesya J, Finocchario-Kessler S, Woldetsadik MA, Khanakwa S, and Wanyenze RK. Correlates of Use of Timed Unprotected Intercourse to Reduce Horizontal Transmission Among Ugandan HIV Clients with Fertility Intentions. AIDS Behav. 2015; 19(6): 1078–1088.

28. Finocchario-Kessler S, Mabachi N, Dariotis JK, Anderson J, Goggin K, Sweat M. “We weren't using condoms because we were trying to conceive”: the need for reproductive counseling for HIV-positive women in clinical care. AIDS Patient Care STDS. 2012;26(11):700-7.

29. Steiner RJ, Dariotis JK, Anderson JR, Finocchario-Kessler S. Preconception care for people living with HIV: recommendations for advancing implementation. AIDS. 2013 Oct;27 Suppl 1:S113-9.

30. Goggin K, Finocchario-Kessler S, Staggs V, Woldetsadik MA, Wanyenze RK, Beyeza-Kashesya J, Mindry D, Khanakwa S, Wagner GJ. Attitudes, Knowledge, and Correlates of Self-Efficacy for the Provision of Safer Conception Counseling Among Ugandan HIV Providers. AIDS Patient Care STDS. 2015; 29(12): 651–660.

31. Mmeje O, Cohen CR, and Cohan1 D. Evaluating Safer Conception Options for HIV-Serodiscordant Couples (HIV-Infected Female/HIV-Uninfected Male): A Closer Look at Vaginal Insemination. Infectious Diseases in Obstetrics and Gynecology. 2012; 587651:7

32. Ngure K, Kimemia G, Dew K, Njuguna N, Mugo N, Celum C, Baeten JM, and Heffron R. Delivering safer conception services to HIV serodiscordant couples in Kenya: perspectives from healthcare providers and HIV serodiscordant couples. J Int AIDS Soc. 2017; 20 Suppl 1: 21309.

33. Uganda National Council for Science and Technology (UNCST). National Guidelines for Research involving Humans. https://uncst.go.ug (2017). Accessed 28 Feb 2018.

Tableau 1 : Femmes de 15 à 49 ans séropositives au VIH prises en charge, en fonction des caractéristiques sélectionnées

Caractéristique	Total, N=5 198 (%)	Région<br>Kampala, N=1 048 (%)	Centre, N=1 032 (%)	Est, N=1 034 (%)	Ouest, N=1 039 (%)	Nord, N=1 045 (%)
**Âge**						
15-19	103 (2,0)	14 (1,3)	22 (2,1)	15 (1,5)	25 (2,4)	27 (2,6)
20-24	657 (12,6)	150 (14,3)	132 (12,8)	115 (11,1)	147 (14,1)	113 (10,8)
25-29	1147 (22,1)	273 (26,0)	209 (20,3)	215 (20,8)	238 (22,9)	212 (20,3)
30-39	2254 (43,4)	452 (43,1)	449 (43,5)	470 (45,5)	437 (42,1)	446 (42,7)
40-49	1037 (20,0)	159 (15,1)	220 (21,3)	219 (21,2)	192 (18,5)	247 (23,6)
**Niveau de l'établissement de santé[1]**						
Hôpital	1556 (29,9)	224 (21,4)	316 (30,6)	284 (27,5)	343 (33,0)	389 (37,2)
CS IV	1540 (29,6)	85 (8,1)	273 (26,5)	405 (39,2)	400 (38,5)	377 (36,1)
CS III	1542 (29,7)	417 (39,8)	366 (35,5)	244 (23,6)	256 (24,6)	259 (24,8)
CS II	416 (8,0)	288 (27,5)	1 (0,1)	81 (7,8)	38 (3,7)	8 (0,8)
Centre de soins privé	112 (2,2)	34 (3,2)	58 (5,6)	20 (1,9)	0 (0,0)	0 (0,0)
Autres	32 (0,6)	0 (0,0)	18 (1,7)	0 (0,0)	2 (0,2)	12 (1,1)
**Religion**						
Catholique	2136 (41,1)	354 (33,8)	441 (42,7)	315 (30,5)	415 (39,9)	611 (58,5)
Anglicane / Protestante	1616 (31,1)	284 (27,1)	273 (26,5)	370 (35,8)	410 (39,5)	279 (26,7)
Musulmane	663 (12,8)	199 (19,0)	150 (14,5)	183 (17,7)	53 (5,1)	78 (7,5)
Pentecôtiste / Chrétienne renaissante / Évangélique	662 (12,7)	188 (17,9)	137 (13,3)	155 (15)	115 (11,1)	67 (6,4)
Autres	121 (2,3)	23 (2,2)	31 (3,0)	11 (1,1)	46 (4,5)	10 (1,0)

**Statut marital**						
Jamais mariée	107 (2,1)	25 (2,4)	7 (0,7)	23 (2,2)	32 (3,1)	20 (1,9)
En couple mais non mariée	1371 (26,4)	349 (33,3)	362 (35,1)	172 (16,6)	163 (15,7)	325 (31,1)
Mariée	3079 (59,2)	596 (56,9)	555 (53,8)	690 (66,7)	672 (64,7)	566 (54,2)
Divorcée/séparée	412 (7,9)	60 (5,7)	77 (7,5)	85 (8,2)	105 (10,1)	85 (8,1)
Veuve	229 (4,4)	18 (1,7)	31 (3)	64 (6,2)	67 (6,4)	49 (4,7)
**Éducation**[2]						
Pas d'éducation	726 (14,0)	65 (6,2)	143 (13,9)	145 (14,0)	157 (15,1)	216 (20,7)
Primaire	2924 (56,3)	449 (42,8)	606 (58,7)	565 (54,6)	653 (62,8)	651 (62,3)
Secondaire	1381 (26,6)	445 (42,5)	260 (25,2)	303 (29,3)	221 (21,3)	152 (14,5)
Post-secondaire	156 (3,0)	89 (8,5)	21 (2)	21 (2,0)	7 (0,7)	18 (1,7)
Non indiquée	11 (0,2)	0 (0,0)	2 (0,2)	0 (0,0)	1 (0,1)	8 (0,8)
**Quintile de richesse**						
Le plus bas	1054 (20,3)	22 (2,1)	170 (16,5)	282 (27,3)	230 (22,1)	350 (33,5)
Deuxième	1026 (19,7)	35 (3,3)	221 (21,4)	244 (23,6)	224 (21,6)	302 (28,9)
Moyen	1041 (20,0)	64 (6,1)	231 (22,4)	253 (24,5)	249 (24,0)	244 (23,3)
Quatrième	1039 (20,0)	328 (31,3)	228 (22,1)	146 (14,1)	239 (23,0)	98 (9,4)
Le plus élevé	1038 (20,0)	599 (57,2)	182 (17,6)	109 (10,5)	97 (9,3)	51 (4,9)
**Possède une radio**						
Non	1822 (35,1)	346 (33,0)	294 (28,5)	399 (38,6)	354 (34,1)	429 (41,1)
Oui	3376 (64,9)	702 (67,0)	738 (71,5)	635 (61,4)	685 (65,9)	616 (58,9)
**Possède un téléphone mobile**						
Non	991 (19,1)	58 (5,5)	187 (18,1)	240 (23,2)	243 (23,4)	263 (25,2)
Oui	4207 (80,9)	990 (94,5)	845 (81,9)	794 (76,8)	796 (76,6)	782 (74,8)
**Sous traitement antirétroviral**						
Non	176 (3,4)	51 (4,9)	6 (0,6)	26 (2,5)	52 (5,0)	41 (3,9)

Oui	5022 (96,6)	997 (95,1)	1026 (99,4)	1008 (97,5)	987 (95,0)	1004 (96,1)
**Durée sous TAR (années)**						
<1	562 (11,3)	166 (16,8)	128 (12,5)	87 (8,7)	100 (10,2)	81 (8,1)
1	570 (11,4)	127 (12,8)	129 (12,6)	105 (10,5)	110 (11,2)	99 (9,9)
2	739 (14,8)	140 (14,1)	157 (15,4)	138 (13,8)	154 (15,7)	150 (15,1)
3+	3118 (62,5)	558 (56,3)	607 (59,5)	670 (67)	617 (62,9)	666 (66,9)
**Statut VIH du partenaire**						
Séropositif	2985 (57,4)	468 (44,7)	539 (52,2)	593 (57,4)	585 (56,3)	800 (76,6)
Séronégatif	954 (18,4)	243 (23,2)	178 (17,3)	201 (19,4)	213 (20,5)	119 (11,4)
Ne sait pas	1259 (24,2)	337 (32,2)	315 (30,5)	240 (23,2)	241 (23,2)	126 (12,1)
**Statut VIH communiqué au partenaire**						
Non	800 (15,5)	301 (29,0)	214 (20,8)	114 (11,1)	133 (12,9)	38 (3,7)
Oui	4362 (84,5)	736 (71,0)	813 (79,2)	914 (88,9)	902 (87,1)	997 (96,3)
**Nombre d'enfants biologiques**						
0	1342 (25,8)	267 (25,5)	318 (30,8)	207 (20,0)	330 (31,8)	220 (21,1)
1	588 (11,3)	193 (18,4)	104 (10,1)	93 (9,0)	104 (10,0)	94 (9,0)
2	852 (16,4)	220 (21,0)	164 (15,9)	133 (12,9)	176 (16,9)	159 (15,2)
3	764 (14,7)	169 (16,1)	132 (12,8)	157 (15,2)	147 (14,1)	159 (15,2)
4+	1652 (31,8)	199 (19,0)	314 (30,4)	444 (42,9)	282 (27,1)	413 (39,5)

[1] Le niveau de l'établissement de santé fait référence au classement des niveaux de soins selon la taille de la population desservie, des niveaux d'effectifs et du type de services offerts, le **CS II** dessert jusqu'à 5 000 personnes et offre uniquement des services ambulatoires, jusqu'à l'hôpital qui offre des soins spécialisés en plus des services

ambulatoires et hospitaliers et des interventions chirurgicales.
[2] Les catégories d'éducation font référence au niveau de scolarisation le plus élevé atteint, qu'il ait été achevé ou non.

Tableau 2 : Connaissance d'au moins une méthode de conception plus sûre en fonction des caractéristiques sélectionnées

Parmi toutes les femmes de 15 à 49 ans, prises en charge pour le VIH, pourcentage ayant répondu « vrai » à au moins une affirmation concernant la connaissance des stratégies de conception plus sûre, classées par caractéristiques socioéconomiques				
	% de femmes non informées	% de femmes informées	Nombre	Valeur P
**Âge**				
15-19	39,8	60,2	103	
20-24	27,4	72,6	657	
25-29	24,5	75,5	1 147	0,014
30-39	25,6	74,4	2 254	
40-49	25,7	74,3	1 037	
**Région**				
Kampala	23,0	77,0	1 048	
Centre	22,7	77,3	1 032	
Est	15,2	84,8	1 034	<0,001
Ouest	34,9	65,1	1 039	
Nord	33,4	66,6	1 045	
**Religion**				
Catholique	27,4	72,6	2 136	
Anglicane/Protestante	25,6	74,4	1 616	
Musulmane	22,0	78,0	663	
Pentecôtiste / Chrétienne renaissante / Évangélique	25,4	74,6	662	0,153
ÉAS*	26,0	74,0	104	
Autre	23,5	76,5	17	
**Sous TAR**				
Non	32,9	67,1	161	0,034
Oui	25,5	74,5	5 022	
Durée sous TAR (années)				
<1	27,7	72,3	559	
1	26,3	73,7	570	0,401
2	26,3	73,7	739	
3	24,7	75,3	3 118	
**Actuellement mariée**				
Non	26,9	73,1	2 119	0,136
Oui	25,1	74,9	3 079	
**Statut marital**				
Jamais mariée	28,0	72,0	107	0,572
En couple mais non mariée	26,5	73,5	1 371	

Mariée	25,1	74,9	3 079	
Divorcée/séparée	28,4	71,6	412	
Veuve	26,2	73,8	229	
**Notification du statut VIH au partenaire**				
Non	26,5	73,5	800	0,634
Oui	25,7	74,3	4 362	
**Statut VIH du partenaire**				
Séropositif	25,6	74,4	2 985	
Séronégatif	23,3	76,7	954	0,017
Ne sait pas/Ne se souvient pas	28,5	71,5	1 259	
**Éducation**				
Pas d'éducation	27,4	72,6	726	
Primaire	26,1	73,9	2 924	
Secondaire	25,0	75,0	1 381	0,574
Post-secondaire	21,8	78,2	156	
Non indiquée	27,3	72,7	11	
**Quintile de richesse**				
Le plus bas	28,9	71,1	1 054	
Deuxième	28,6	71,4	1 026	
Moyen	25,1	74,9	1 041	0,003
Quatrième	23,3	76,7	1 039	
Le plus élevé	23,4	76,6	1 038	
**Niveau de l'établissement de santé**				
Hôpital	26,5	73,5	1 556	
Centre de santé IV	26,5	73,5	1 540	
Centre de santé III	26,2	73,8	1 542	0,008
Centre de santé II	23,6	76,4	416	
Centre de soins privé	10,7	89,3	112	
Autres	30,8	69,2	26	
**Propriétaire**				
Gouvernement/Secteur public	26,1	73,9	3 575	
Mission/ONG/Privé à but non lucratif	25,3	74,7	1 549	0,784
Privé à but lucratif	23,9	76,1	71	
**Total**	**25,9**	**74,1**	**5 198**	
***ÉAS fait référence aux membres de l'Église adventiste du septième jour**				

Tableau 3 : Connaissance de méthodes de conception plus sûres spécifiques

Parmi toutes les femmes de 15 à 49 ans, prises en charge pour le VIH, pourcentage ayant répondu « vrai » aux affirmations concernant des stratégies spécifiques de conception plus sûre, classées par caractéristiques socioéconomiques						
	Femmes qui savent que :					
Caractéristiques socioéconomiques	Avoir une charge virale « indétectable » réduit le risque de transmission du VIH.	Si l'homme éjacule dans un préservatif ou un récipient puis injecte manuellement le sperme dans le vagin de la femme, ceci permet de réduire le risque de transmission du VIH si l'homme est séronégatif au VIH (AA)	Avoir des rapports non protégés pendant les jours du mois où une femme est plus fertile aidera à limiter le risque de transmission du VIH à un partenaire non infecté » (RNPP)	Il existe des techniques permettant de laver le sperme de l'homme pour éliminer le virus du VIH (lavage du sperme)	Les traitements contre le VIH peuvent être pris par un partenaire séronégatif (ou dont le statut est inconnu) pour réduire leur risque d'être infecté par leur partenaire VIH+ (PrEP)	Nombre de femmes
**Âge**						
15-19	26,7	24,8	31	16,8	27,6	103
20-24	32,2	34,2	37,5	16,3	22,5	657
25-29	35,1	36,4	40,1	15,6	26,4	1 147
30-39	34,3	35,7	39,7	19	24,4	2 254
40-49	33,2	32,6	38,1	18,8	25,1	1 037
**Statut marital**						
Jamais mariée	34,9	37,7	32,1	18,9	29,5	107

En couple mais non mariée	36,7	32,9	38,1	17,3	23,2	1 371
Mariée	33,6	35,3	39,9	18	25,2	3 079
Divorcée/séparée	29,5	35,5	38,8	19,1	26,8	412
Veuve	26,9	36,8	36,4	16,3	23,5	229
**Région**						
Kampala	41,4	25,3	45,6	19,6	25,8	1 048
Centre	43,3	30,8	46,9	18,4	17,4	1 032
Est	33	62,5	38	29,2	37	1 034
Ouest	21,9	30,4	33,3	7,2	18,9	1 039
Nord	29,6	25,2	31,4	14,8	24,8	1 045
**Éducation**						
Pas d'éducation	30,5	31,5	40,4	18,9	23,1	726
Primaire	33,5	34,5	39,9	17,6	24,1	2 924
Secondaire	35,5	37,2	36,9	17,7	26,9	1 381
Post-secondaire	39,7	36,5	36,5	19,9	27,1	156
Non indiquée	63,6	18,2	18,2	9,1	36,4	11
**Quintile de richesse**						
Le plus bas	30,3	34,8	33,3	16	24,1	1 054
Deuxième	32,5	34,9	37,2	17,1	23,6	1 026
Moyen	33,9	37,2	41,3	18,5	25,7	1 041
Quatrième	35,4	34,7	41,6	18,3	25,1	1 039
Le plus élevé	37,2	32,4	41,7	19,3	25,5	1 038
Total	33,8	34,8	39,0	17,8	24,8	5 198

Tableau 4. Corrélations de la connaissance des méthodes de conception plus sûres

Répartition des femmes, prises en charge pour le VIH et âgées de 15 à 49 ans, qui ont indiqué connaître une méthode de conception plus sûre (RNPP, AA, PrEP), classées en fonction des caractéristiques socioéconomiques sélectionnées				
	**Bivariée**		**Multivariée**	
Caractéristiques socioéconomiques	RP (IC 95 %)	valeur p	RP (IC 95 %)	valeur p
**Âge**				
15-19	Réf.			
20-24	1,206 (1,024, 1,421)	0,025	1,150 (0,977, 1,354)	0,093
25-29	1,254 (1,068, 1,473)	0,006	1,188 (1,011, 1,395)	0,036
30-39	1,237 (1,055, 1,449)	0,009	1,168 (0,996, 1,371)	0,056
40-49	1,235 (1,051, 1,451)	0,010	1,165 (0,990, 1,371)	
**Région**				
Nord	Réf.			
Kampala	1,156 (1,095, 1,220)	0,000	1,138 (1,064, 1,218)	0,000
Centre	1,161 (1,099, 1,226)	0,000	1,136 (1,072, 1,203)	0,000
Est	1,273 (1,211, 1,339)	0,000	1,256 (1,191, 1,324)	0,000
Ouest	0,977 (0,918, 1,039)	0,459	0,956 (0,896, 1,019)	0,172
**Religion**				
Catholique	Réf.			
Anglicane/Protestante	1,027 (0,987, 1,066)	0,196	1,011 (0,972, 1,051)	0,583
Musulmane	1,074 (1,024, 1,127)	0,003	1,016 (0,967, 1,067)	0,526
Pentecôtiste / Chrétienne renaissante / Évangélique	1,028 (0,977, 1,083)	0,288	0,994 (0,944, 1,047)	0,834
Église adventiste du septième jour	1,020 (0,908, 1,147)	0,736	1,034 (0,919, 1,162)	0,576
Autre	1,054 (0,808, 1,373)	0,698	1,108 (0,832, 1,474)	0,482
**Sous TAR**				
Non	Réf.			
Oui	1,110 (0,995,	0,061		

	1,239)			
Durée sous TAR (années)				
<1	Réf.			
1	1,019 (0,949, 1,094)	0,593		
2	1,020 (0,954, 1,091)	0,554		
3	1,042 (0,986, 1,101)	0,144		
**Actuellement mariée**				
Non	Réf.			
Oui	1,025 (0,992, 1,059)	0,139		
**Statut marital**				
Jamais mariée	Réf.			
En couple mais non mariée	1,021 (0,903, 1,154)	0,743		
Mariée	1,041 (0,923, 1,173)	0,514		
Divorcée/séparée	0,995 (0,871, 1,136)	0,941		
Veuve	1,025 (0,890, 1,181)	0,727		
**Notification du statut VIH au partenaire**				
Non	Réf.			
Oui	1,011 (0,966, 1,057)	0,638		
**Statut VIH du partenaire**				
Séropositif	Réf.			
Séronégatif	1,031 (0,989, 1,074)	0,145	1,018 (0,977, 1,061)	0,395
Ne sait pas/Ne se souvient pas	0,960 (0,922, 1,000)	0,051	0,957 (0,911, 1,006)	0,083
**Éducation**				
Pas d'éducation	Réf.			
Primaire	1,018 (0,969, 1,069)	0,478		
Secondaire	1,033 (0,979, 1,091)	0,233		
Post-secondaire	1,077 (0,980, 1,184)	0,121		
Non indiquée	1,002 (0,696,	0,992		

	1,443)			
**Quintiles de richesse**				
Le plus bas	Réf.			
Deuxième	1,005 (0,952, 1,062)	0,848	1,016 (0,961, 1,073)	0,578
Moyen	1,054 (1,001, 1,111)	0,047	1,062 (1,007, 1,119)	0,027
Quatrième	1,079 (1,026, 1,136)	0,003	1,089 (1,031, 1,152)	0,003
Le plus élevé	1,078 (1,024, 1,134)	0,004	1,055 (0,992, 1,122)	0,090
**Niveau de l'établissement de santé**				
Hôpital	Réf.			
Centre de santé IV	1,001 (0,959, 1,044)	0,975	1,002 (0,958, 1,048)	0,939
Centre de santé III	1,005 (0,963, 1,048)	0,829	0,994 (0,952, 1,039)	0,801
Centre de santé II	1,041 (0,979, 1,106)	0,202	0,962 (0,898, 1,031)	0,270
Centre de soins privé	1,215 (1,132, 1,305)	0,000	1,142 (1,058, 1,234)	0,001
Autres	0,942 (0,728, 1,219)	0,653	0,966 (0,748, 1,248)	0,790
**Propriétaire**				
Gouvernement/Secteur public	Réf.			
Mission/ONG/Privé à but non lucratif	1,011 (0,976, 1,047)	0,550		
Privé à but lucratif	1,029 (0,902, 1,174)	0,670		

Tableau 5 : Utilisation de méthodes de conception plus sûres

Répartition en pourcentage des femmes, prises en charge pour le VIH et âgées de 15 à 49 ans, qui ont indiqué avoir utilisé une méthode de conception plus sûre (RNPP, AA, PrEP) pour réduire la transmission du VIH, classées en fonction des caractéristiques socioéconomiques sélectionnées

Caractéristiques socioéconomiques	Pourcentage, %	Nombre de femmes
**Âge**		
15-19	14,0	43
20-24	12,4	355
25-29	11,5	529
30-39	23,6	744
40-49	19,5	125
**Région**		
Kampala	8,2	355
Centre	13,8	290
Est	4,6	395
Ouest	13,7	424
Nord	19,3	332
**Religion**		
Catholique	11,2	723
Anglicane / Protestante	14,1	560
Musulmane	9,0	256
Pentecôtiste / Chrétienne renaissante / Évangélique	11,2	215
Autres	19,5	42
**Statut marital**		
Jamais mariée	5,0	20
En couple mais non mariée	10,6	396
Mariée	12,2	1 246
Divorcée/séparée	12,1	99
Veuve	5,7	35
**Éducation**		
Pas d'éducation	11,6	224
Primaire	12,3	1 028
Secondaire	10,2	491
Post-secondaire	14,0	50
Non indiquée	0,0	3
**Connaissance générale de l'existence de la conception plus sûre**		
Non	7,0	371
Oui	12,9	1 425

**Connaissance d'une des méthodes de conception plus sûre**		
Non	11,8	601
Oui	11,5	1 195
**Catégories de connaissances de la conception plus sûre**		
Connaissait une seule méthode (niveau de connaissance bas)	11,8	1 394
Connaissait deux méthodes (niveau de connaissance moyen)	13,2	243
Connaissait 3 à 4 méthodes (niveau de connaissance élevé)	8,6	159
**Total 15-49**	**11,6**	**1 796**

Tableau 6. <u>Corrélations de l'utilisation de méthodes de conception plus sûres</u>

Répartition des femmes, prises en charge pour le VIH et âgées de 15 à 49 ans, qui ont indiqué avoir utilisé une méthode de conception plus sûre (RNPP, AA, PrEP) pour réduire la transmission du VIH, classées en fonction des caractéristiques socioéconomiques sélectionnées

	Bivariée		Multivariée	
Caractéristiques socioéconomiques	RP (IC 95 %)	valeur p	RP (IC 95 %)	valeur p
**Âge**				
15-19	Réf.			
20-24	0,888 (0,402, 1,961)	0,769		
25-29	0,826 (0,379, 1,801)	0,631		
30-39	0,809 (0,375, 1,746)	0,589		
40-49	0,803 (0,329, 1,958)	0,629		
**Région**				
Nord	Réf.		Réf.	
Kampala	0,424 (0,281, 0,640)	0,001	0,489 (0,314, 0,764)	0,002
Centre	0,716 (0,498, 1,028)	0,070	0,818 (0,567, 1,179)	0,282
Est	0,236 (0,143, 0,391)	0,001	0,244 (0,147, 0,405)	0,001
Ouest	0,709 (0,513, 0,982)	0,039	0,726 (0,518, 1,017)	0,063
**Niveau de l'établissement de santé**				
Hôpital	Réf.		Réf.	
Centre de santé IV	1,076 (0,769, 1,506)	0,667	1,159 (0,828, 1,622)	0,390
Centre de santé III	1,136 (0,818, 1,578)	0,446	1,274 (0,917, 1,772)	0,149
Centre de santé II	0,736 (0,386, 1,402)	0,351	1,143 (0,579, 2,254)	0,700
Centre de soins privé	0,249 (0,036, 1,752)	0,163	0,301 (0,041, 2,213)	0,238
Autres	3,849 (1,579, 9,385)	0,003	2,578 (1,001, 6,635)	0,050
**Religion**				
Catholique	Réf.			
Anglicane / Protestante	1,259 (0,942, 1,682)	0,119		
Musulmane	0,802 (0,516, 1,246)	0,326		
Pentecôtiste / Chrétienne renaissante / Évangélique	0,996 (0,649, 1,531)	0,987		
Église adventiste du septième jour	0,248 (0,035, 1,732)	0,160		
Autres	1,487 (0,246, 9,013)	0,666		

**TAR**				
Non	Réf.			
Oui	3,775 (0,546, 26,110)	0,178		
**Durée sous TAR**				
0	Réf.			
1	1,325 (0,767, 2,288)	0,313		
2	1,479 (0,874, 2,501)	0,144		
3+	1,474 (0,929, 2,338)	0,100		
**Statut marital**				
Mariée	Réf.			
Non mariée	1,177 (0,883, 1,569)	0,266		
**Statut VIH communiqué au partenaire**				
Non	Réf.		Réf.	
Oui	3,416 (1,776, 6,570)	0,001	2,613 (1,308, 5,221)	0,007
**Statut VIH du partenaire**				
Séropositif	Réf.		Réf.	
Séronégatif	1,426 (1,076, 1,889)	0,013	1,637 (1,236, 2,168)	0,001
Ne sait pas	0,583 (0,392, 0,866)	0,008	0,906 (0,595, 1,380)	0,646
**Statut TAR du partenaire**				
Oui	Réf.			
Non	1,408 (0,812, 2,439)	0,223		
Ne sait pas	0,685 (0,227, 2,063)	0,501		
**Éducation**				
Pas d'éducation	Réf.			
Primaire	1,056 (0,710, 1,570)	0,788		
Secondaire	0,877 (0,561, 1,372)	0,566		
Post-secondaire	1,206 (0,555, 2,622)	0,636		
**Nombre d'enfants biologiques**				
0	Réf.			
1	1,086 (0,683, 1,726)	0,727		
2	1,047 (0,694, 1,581)	0,826		
3	0,685 (0,416, 1,128)	0,137		
4+	1,096 (0,756, 1,589)	0,629		
**Enceinte (grossesse souhaitée) ou prévoit d'avoir un enfant dans**				

**les deux ans**				
Oui	Réf.			
Non	1,102 (0,768, 1,583)	0,597		
**Connaissance générale de l'existence de la conception plus sûre**				
Non	Réf.		Réf.	
Oui	1,838 (1,238, 2,727)	0,03	2,953 (1,968, 4,430)	<0,001
**Connaissance d'une des méthodes de conception plus sûre**				
Non	Réf.			
Oui	0,977 (0,747, 1,279)	0,868		
**Catégories de connaissances de la conception plus sûre**				
Connaissait une seule méthode	Réf.			
Connaissait deux méthodes	1,126 (0,798, 1,590)	0,497		
Connaissait trois à quatre méthodes	0,733 (0,461, 1,168)	0,192		
