# Supplementary material for: Knowledge and correlates of use of safer conception methods among HIV-infected women attending HIV care in Uganda
Source: Reprod Health. 2019 May 29;16(Suppl 1):64. doi: 10.1186/s12978-019-0717-6 (PMC6538556; doi:10.1186/s12978-019-0717-6)
Supplement: Supplementary file 2 — Translation of the abstract of this article into Portuguese (PDF 101 kb) [file 12978_2019_717_MOESM2_ESM.pdf]

Correlação do conhecimento e do uso de métodos de conceção mais seguros entre mulheres seropositivas (VIH) a receber cuidados de saúde para o VIH no Uganda

Violet Gwokyalya[1*], Jolly Beyeza-Kashesya[2], John Baptist Bwanika[1], Joseph KB Matovu[1], Shaban Mugerwa[3], Jim Arinaitwe[3], Dickson Kasozi[1], Justine Bukenya[1], Rosemary Kindyomunda[4], Glenn J. Wagner [5], Fredrick E Makumbi[1] and Rhoda K. Wanyenze[1]

Afiliações: [1]Makerere University School of Public Health, Kampala, Uganda, [2]Department of Obstetrics and Gynecology, Mulago National Referral Hospital, Kampala, Uganda, [3]Ministry of Health, Kampala, Uganda, [4]UNFPA, Kampala, Uganda, [5]RAND Corporation, Santa Monica, CA, USA.

***Autor correspondente:**

Violet Gwokyalya: vgwokyalya@musph.ac.ug

Endereço de e-mail do autor:

Jolly Beyeza-Kashesya:	jbeyeza@gmail.com
John Baptist Bwanika:	bwanikajb@yahoo.com
Joseph KB Matovu:	jmatovu@musph.ac.ug
Shaban Mugerwa:	shabanmugerwa@gmail.com
Jim Arinaitwe:	jim_ari@yahoo.co.uk
Dickson Kasozi:	dikasozi@gmail.com
Rosemary Kindyomunda:	kindyomunda@unfpa.org
Justine Bukenya:	jbukenya@musph.ac.ug
Glenn J. Wagner:	gwagner@rand.org
Fredrick E Makumbi:	fmakumbi@musph.ac.ug
Rhoda K. Wanyenze:	rwanyenze@musph.ac.ug

Resumo

Introdução: Muitas pessoas portadoras de VIH gostariam de ter filhos, mas os serviços de planeamento familiar (PF) centram-se frequentemente na contraceção. A disponibilidade de serviços de conceção mais seguros ainda é muito baixa na maioria dos países de baixo rendimento. Neste estudo, foi avaliado o conhecimento e uso de métodos de conceção mais seguros entre as mulheres infetadas com VIH a receber cuidados de saúde para o VIH no Uganda, para informar sobre a integração de métodos de conceção mais seguros nos serviços de PF existentes.

Métodos: Os dados foram recolhidos a partir de um inquérito transversal de representatividade nacional com 5198 mulheres entre 15 e 49 anos infetadas com o VIH em 245 clínicas de VIH no Uganda. O conhecimento e uso de métodos de conceção mais seguros, incluindo outros fatores associados, também foram determinados. A medida de associação utilizada foi a taxa de prevalência (TP) com base em intervalos de confiança a 95%, obtidos por meio da regressão de Poisson modificada através de modelos lineares generalizados. Todas as análises foram realizadas com o software STATA (versão 12.0).

Resultados: O conhecimento global sobre qualquer método de conceção mais seguro foi de 74,1% (3852/5198). Contudo, apenas 13,2% tinha conhecimento de três dos quatro métodos, 18,9% tinha conhecimento de apenas dois métodos e 42% apenas tinha conhecimento de um método. O conhecimento de métodos de conceção mais seguros específicos foi superior para um período de tempo de relações sexuais sem proteção com 39% (n=2027), seguido de autoinseminação manual com 34,8% (n=1809) e profilaxia de pré-exposição com 24,8% (n=1289). O conhecimento de métodos de conceção mais seguros foi superior na região leste (84,8%, p<0,001), entre mulheres em relações serodiscordantes para VIH (76,7%, p<0,017) e aquelas a receber TAR (74,5%, p<0,034). No geral, 1796 mulheres (34,6%) estavam grávidas ou registaram um nascimento nos últimos dois anos, o que significa que a utilização global de métodos de conceção mais seguros neste grupo foi de 11,6% (209/1796). A probabilidade do uso de métodos de conceção mais seguros foi substancialmente inferior em Campala [TP ajustada= 0,489 (0,314; 0,764)] ou na região leste [TP ajustada=0,244 (0,147; 0,405)], em comparação com a região norte. A maior probabilidade do uso de métodos de conceção mais

seguros foi associada à divulgação do estatuto de portador de VIH ao parceiro [TP ajustada= 2,613 (1,308; 5,221)] e a relações serodiscordantes em comparação com relações seroconcordantes para VIH [TP ajustada=1,637(1,236; 2,168)]. O conhecimento pré-existente dos métodos de conceção mais seguros não influenciou a utilização dos mesmos.

Conclusões: O conhecimento e uso de métodos de conceção mais seguros entre as mulheres seropositivas a receber cuidados de saúde é reduzido. Os esforços para melhorar a divulgação do estatuto de portador de VIH e a integração de métodos de conceção mais seguros nos serviços de PF e VIH e os esforços regionais para promover a sensibilização e o acesso a métodos de conceção mais seguros podem contribuir para aumentar a adoção de métodos de conceção mais seguros.

Palavras-chave: Métodos de conceção mais seguros, Conhecimento, VIH, Uganda, Saúde reprodutiva

Sobre este suplemento

Este resumo foi publicado como parte da revista científica *Reproductive Health*, Volume 16, Suplemento 1, 2019: Integração Eficaz dos Serviços de Saúde Sexual e Reprodutiva e de Prevenção, Cuidados e Tratamento do VIH na África Subsariana: Onde estão as provas da implementação do programa?
O suplemento foi publicado como uma colaboração entre as revistas científicas *Reproductive Health* e *BMC Public Health.* O conteúdo integral, incluindo as versões em francês, português e inglês, estão disponíveis online:
https://bmcpublichealth.biomedcentral.com/articles/supplements/volume-19-supplement-1
e
https://reproductive-health-journal.biomedcentral.com/articles/supplements/volume-16-supplement-1
